# Discovering Human Activities from Binary Data in Smart Homes

**DOI:** 10.3390/s20092513

**Published:** 2020-04-29

**Authors:** Mohamed Eldib, Wilfried Philips, Hamid Aghajan

**Affiliations:** imec-TELIN-IPI, Ghent University, Sint-Pietersnieuwstraat 41, 9000 Ghent, Belgium; Wilfried.Philips@UGent.be (W.P.); Hamid.Aghajan@UGent.be (H.A.)

**Keywords:** human activity discovery, smart homes, health monitoring, clustering, unsupervised learning, sequence mining, frequent patterns

## Abstract

With the rapid development in sensing technology, data mining, and machine learning fields for human health monitoring, it became possible to enable monitoring of personal motion and vital signs in a manner that minimizes the disruption of an individual’s daily routine and assist individuals with difficulties to live independently at home. A primary difficulty that researchers confront is acquiring an adequate amount of labeled data for model training and validation purposes. Therefore, activity discovery handles the problem that activity labels are not available using approaches based on sequence mining and clustering. In this paper, we introduce an unsupervised method for discovering activities from a network of motion detectors in a smart home setting. First, we present an intra-day clustering algorithm to find frequent sequential patterns within a day. As a second step, we present an inter-day clustering algorithm to find the common frequent patterns between days. Furthermore, we refine the patterns to have more compressed and defined cluster characterizations. Finally, we track the occurrences of various regular routines to monitor the functional health in an individual’s patterns and lifestyle. We evaluate our methods on two public data sets captured in real-life settings from two apartments during seven-month and three-month periods.

## 1. Introduction

The steady increase in our older adult population, coupled with older adults living longer and the requirement for more assistance, is driving caregiver demand. According to the United Nations’ World Population Prospects report, the number of people over the age of 60 is expected to be more than doubled by 2050 and more than tripled by 2100. Further, all major areas of the world will have nearly a quarter or more of their populations be 60 or over [1]. This puts burdens on formal and informal caregivers to provide care for an average of 20 h per week [2] for a chronically ill, disabled or aging family member or friend. Besides the demands of care-giving, there is pressure on the economic side to support the healthcare sector; the EU countries spend on average a quarter of their GDP on social protection [3]. This highlights the needs for effective solutions to tackle the demographic and economic challenges towards a better health and social care of the older population.

Ambient assisted living (AAL) provides one of the most promising solutions with which to support the older people who wish to keep their independence as long as possible, trying to fend for themselves by having fulfilling lives in terms of everyday activities and leisure. With the introduction of new low-cost sensors, low-power wireless protocols, cloud computing, and Internet of Things (IoT) technologies, the ability to deploy tools in our daily lives has never been more available. Assisted-care and health-monitoring became more available in order to contentiously observe and keep an eye on the behaviors of the senior adults. Activities of daily living (ADLs) is a term used to represent the set of common tasks that comprise a person’s everyday requirements that influence the self-support of a senior adult in day-to-day routines. The essential list of ADLs include but are not limited to relaxing, meal preparation, eating, and sleeping. The power to perform ADLs without assistance from the others and in a constant manner provides a necessary assessment of the functional condition of the senior adult and the power to live without support at home. In a recent survey [4], formal and informal caregivers showed an increasing interest in an accurate activity recognition and tracking.

In this paper, we use a smart environment infrastructure to observe the routines of senior adults [5,6,7,8,9]. A smart environment is equipped with various kinds of sensors, such as infrared motion sensors (PIR), for monitoring the motions of the occupant at home. Such sensors can collect many representative details about the surroundings and its occupants. There are several potential applications for a smart environment, such as recommendation in office environments, energy preservation, home automation, surveillance, and of course assisted care of senior adults [10,11]. Activity learning and tracking are some of the core components of any smart environment for ADL monitoring. The purpose of an activity learning component is to identify the inhabitant activities from the low-level sensor data. The various proposed approaches in the literature include a number of large differences with respect to the primary sensing technology, the artificial intelligence approaches, and the practicality of the smart homes in which activity information is captured.

However, modeling all human activities in a supervised-based approach faces a number of challenges and obstacles. First, a large amount of sensor data must be available. The sensor data should be labeled with the actual activities (the “ground truth” labels). In real-world in-home monitoring systems, such prelabeled data are very difficult to obtain. Second, the time that is spent on activities, which are easy to annotate (e.g., sleep times), is only a fraction of an individual’s total behavioral routine. Beside that, modeling and tracking only preselected activities ignores the important insights that other activities can provide on the routine behavior and the activity context of the individual. Finally, it is very challenging to build an unintrusive sensing system to collect the data from real individuals, annotate the data with the ground truth label for training and testing purposes, and develop efficient methods to learn and accurately detect the activities. Therefore, activity discovery handles the problem of activity labels not being available using approaches based on sequence mining and clustering. Not only that, activity discovery methods can work better with mining human activities from real-life data, given its natural sequence, the continuation of disarranged patterns, and the different forms of the same pattern [5].

The work in this paper regards activity learning on discrete, binary motion sensor data collected from real-world environments. We are mainly interested in answering questions related to the use of motion sensors. “Can the activities be automatically discovered (learned) by looking for interesting patterns in the data from the motion sensors without relying on supervised methods?” Data annotation is a very time consuming and laborious task. It is very difficult to obtain labels for all the activities in the data set. Even with the assumption of consistent predefined activities, not all individuals perform them the same way due to the complex structure of activities. Therefore, supervised methods are not practical to use. We introduce an unsupervised activity discovery algorithm that does not depend on any activity annotations in the data set.

Our framework consists of three processing steps; namely, low-level, intermediate-level, and high-level units. In the low-level processing step, the raw sensor data is preprocessed to be sampled periodically at a constant time interval. In the intermediate-level step, we use a time-based windowing approach to divide the sensor data into time intervals using two different time interval lengths. First, we use a small time interval length to extract the time duration within the detection area of each sensor. Next, we use a large time interval length to extract the time duration within the detection of each location. The small and large time intervals are processed to extract meaningful features.

In the high-level step, we introduce several clustering algorithms and approaches to form clusters based on the statistical features encapsulated in the different time intervals. First, we perform an intra-day pattern discovery where we group similar time intervals to form the clusters within the same day using an exponential function as a similarity measure between the different time intervals. Next, we perform an inter-day pattern discovery, where we group similar clusters across all days to ensure the uniqueness of the clusters using the Earth mover’s distance (EMD) as a similarity measure between the clusters. Furthermore, the clusters are refined to have more compressed and defined characterizations. Finally, the clusters are given semantic labels in order to track the occurrences of the future activities.

We discuss two approaches for recognizing activities using the final set of the clusters, where they are used to recognize the occurrences of the future activities from a window of sensor events. The first approach uses a model to classify the labels, while the second approach measures the similarity between the clusters’ centroids, and the test time interval within which to determine the cluster with the best similarity value, where the semantic label of that cluster is assigned. Our method handles other real-life difficulties, such as major various sensor event occurrences in different areas of the home. It completely removes the requirement to set most of the system values by the occupant, yielding to a more system-driven approach. This framework is evaluated on two public data sets captured in real-life settings from two apartments during a seven-month and a three-month periods. The data sets we use reflect the challenges of unrestricted real-life data, measured when the user is functioning in their natural home and performing their day-to-day activities with no directions or orders from the scientists.

This paper is organized as follows. Section 2 describes the related work. In Section 3, we introduce our proposed framework. In Section 4, we illustrate the steps taken to preprocess the raw sensor data. In Section 5, we discuss our approach to extract meaningful features from the raw sensor data. In Section 6, we introduce different clustering approaches and algorithms to form clusters based on the statistical features encapsulated in the different time intervals of the sensor events. Activity recognition is presented in Section 7. We present and discuss the experimental results in Section 8. Finally, Section 9 draws conclusions.

## 2. Related Work

A large number of works chose event-like sensors as their primary sensing technologies for data capturing, because they are computationally inexpensive, easy to install, cheap, privacy-friendly in nature, long in battery life, do not have to be carried or worn, and require minimal supervision and maintenance. This led to generating many public data sets based on event-like sensors for long duration measurements with rich information. There are still some challenges in the use of event-like sensors, such as passive infrared (PIR) sensors. First, PIR sensors produce highly bursty output, which limits PIR systems to single-person scenarios, and self-triggering due to sudden changes in environmental conditions, such as heating, ventilating, and air conditioning. Furthermore, PIR sensors may not be able to sense all motions if an obstacle is between the sensor and the moving person. Moreover, a movement can be sensed by a PIR sensor from any object that emits heat, even if the movement comes from an inorganic source. As a result, events may be generated by the motion sensor, when a printer starts printing documents in large quantities in the vicinity or if a close by baseboard heater suddenly switches on.

There are many proposed approaches toward recognizing the activities of daily living in a home setting with PIR sensors, which can be broadly categorized into two major categories: supervised and unsupervised approaches [12,13,14,15].

Among these two categories, supervised approaches are much more prevalent than unsupervised, since the task of recognizing activities of daily living can be easily formatted into a classification problem, which can be solved by a lot of mature classification algorithms. By using the sequence of PIR sensor events with their corresponding time and location information as input features, and the activity labels as ground truth, many popular machine learning algorithms, such as the naive Bayesian classifier [16,17], decision trees [18], a support vector machine (SVM) [19], and a neural network [20], can be directly applied to activity recognition tasks [21]. Additionally, kernel fusion is a favorable approach with which to obtain accurate results. A kernel fusion approach based on an SVM classifier is proposed in [22], wherein the authors used four individual SVM kernel functions, wherein each kernel was designed to learn the activities in parallel. Additionally, the use of XAI models [23] can contribute further to explaining the machine learning model results, and provide conclusions on these model behaviors and decisions about the human activity recognition problem.

Moreover, template matching methods such as k-nearest neighbors, which can be either based on Euclidean distance using location information in the sensor data or rely on edit distance with sequence details of the sensor activation events, have also been proposed for the activity recognition problem [18,24]. In [25], the authors proposed a modified machine learning algorithm based on k-nearest neighbors (MkRENN) for activity recognition. In addition, because of the sequential nature of the PIR sensor data, the hidden Markov model (HMM), hierarchical hidden semi-Markov models, dynamic Bayesian networks, and many other popular graphical models have been used to model the activity transition sequence for activity recognition purposes [24,26,27,28,29,30,31].

In [32], the author explored the use of a semi-supervised machine learning algorithm to teach generalized setting activity models. The author constructed an ensemble classifier wherein the naive Bayes classifier, HMM, and conditional random field (CRF) models were the base classifiers of this ensemble, and a boosted decision tree was a top classifier. Then, a semi-supervised learning method was used to teach the final model. The final model was trained on 11 data sets. In [33], the authors followed a similar approach, in which they constructed a neural network ensemble. A method for activity recognition is proposed in [34] by optimizing the output of multiple classifiers with the genetic algorithm (GA). The different measurement level outputs of different classifiers, such as HMM, SVM, and CRF, were combined to form the ensemble.

Even though the majority of the surveyed activity recognition approaches are supervised methods, most of them share the same limitation: (1) the requirement for sufficient amount of labeled data in order to work well; and (2) accurate activity labels for PIR sensor data sets. For almost all of the current smart home test-beds with PIR sensors, the data collection and data labeling are two separate processes, among which the activity labeling for the collected PIR sensor data is extremely time consuming and laborious because it is usually based on direct video coding and manually labeling. Clearly, this limitation prevents the supervised approaches from being easily generalized to a real-world situation, wherein activity labels are usually not available for a huge amount of sensor data.

Intuitively, activities of daily living are mostly the occupant’s daily or weekly routines. They usually follow some similar sequences and repeat a lot, which means the interleaving connectivity and periodic or frequent patterns within the raw PIR sensor data themselves can be very useful for activity discovery and recognition purposes. Therefore, a number of unsupervised approaches have been proposed to handle the problem of activity labels not being available. Many of them are based on sequence mining algorithms, such as frequent sequence mining approaches that use different sliding windows to find frequent patterns [35,36,37]; and the emerging pattern mining also known as contrast pattern-mining [38,39,40] that uses significant changes and differences in the data set as features to differentiate between different activities [41,42,43]. Gu et al. [41] used the notion of emerging patterns to look for common sensor sequences that can be linked to each activity as a support for recognition.

In [44], the author analyzed the sensor data at the location level only, and constructed a feature vector of one dimension with time series information. Xie et al. [45] proposed a hybrid system that used an inertial sensor and a barometer to learn static activities and detect locomotion. The authors in [46] proposed an approach based on zero-shot learning to learn human activities. Hamad et al. [47] proposed a fuzzy windowing approach to extract temporal features for human activity recognition. Müller et al. [48] studied several unsupervised learning methods using sensor data streams from multiple residents. Fang et al. [49] proposed a method that depends on the integration of several hierarchical mixture models of directional statistical models with active learning strategies to build an online and active learning system for activity learning. The authors in [50] presented a method that can track activities and detect deviations from long-term behavioral routines using binary sensors.

Some of the recent sequence mining approaches are presented in [5,51], wherein the authors proposed two methods; namely, discontinuous varied-order sequence mining (DVSM) and continuous varied order (COM) which can automatically discover discontinuous patterns with different event orders across their instances. In the first approach, DVSM worked best when using data collected under controlled conditions and in artificial settings. It faced difficulty mining real-life data. The DVSM method was able to discover only five activities out of ten predefined activities. In the second approach, COM was an improved version of DVSM. COM was also able to better handle real-life data by dealing with the different frequencies problem. Additionally, it was able to find a higher percentage of the frequent patterns, thereby achieving a higher recognition accuracy rate. The COM method was successfully able to discover only seven activities out of ten predefined activities. It can be noted that both methods were not able to discover all predefined activities. These methods were not able to deal with some similar activities and might mistake them with one another. Some other activities such as housekeeping were not discovered in the first place.

Other approaches include the graph-based pattern discovery method, which discovers possible activities by searching for sequence patterns that best compress the input unlabeled data [52,53]; activity learning and recognition based on multiple eigenspaces [54]; activity episode mining that identifies significant episodes in sequential data by evaluating the significance values of the candidates [14,55]; and the hierarchical modeling method which models the occupant’s activity at different levels, such as activity, movements, and motion from top to bottom, so that level by level clustering and classification can be applied to reduce the difficulty of the overall activity discovery and recognition [10]. In addition, techniques based on graph analysis have also been proposed to analyze and discover the occupant’s activity using PIR sensor network data sets [15,56,57,58].

The surveyed approaches consider a more simple case of the problem by not fully analyzing the real-world nature of data, such as their order or sequence, the probable interruptions (e.g., a toilet visit during a sleep time), or the differences between the same patterns. Moreover, not all the labeled activities were discovered and recognized. Differently from the standard sequence mining methods, the diverse activity patterns are expected to be detected from a continuous stream of binary data with no explicit partitioning between two following activities. As a consequence, the sensor data that represent an activity pattern can have high variations in their lengths. These problems present major challenges in discovering human activities from binary sensor data, where the sequence of the binary data can be stopped or interrupted by minor events, and can have different forms with regard to the order of the sequence, its length, its time duration, and its occurrence in specific locations.

Our method attempts to find general recurring patterns as well as pattern variations taking into consideration the erratic nature and varying order of the human activities. Our method has several advantages over the other methods. First, we use several time interval lengths in order to capture several cues and important features in the sensor data. This enables our method to handle varying frequencies for activities performed in different locations by keeping only relevant variations of patterns, and pruning other irrelevant variations. Second, we use the location as a cue in our clustering algorithms. Many of the aforementioned methods do not use this cue. The location as a cue significantly boosts the accuracy in our methods, and it has shown promising results in human activity discovery [5]. Third, we rely on several cluster properties to measure the similarity between clusters, and this ensures a better cluster quality. Finally, our clustering algorithms eliminate the need for the user to configure the number of clusters. This results in a more automated approach overall.

## 3. Our Proposed Framework

**Figure 1:**
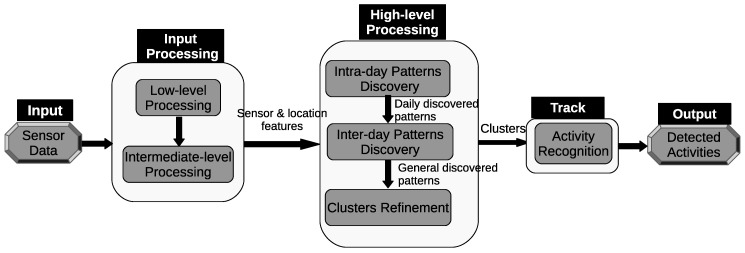
Main components of our proposed framework.

The architecture of the proposed framework can be seen in Figure 1. The first component consists of one “low-level processing” unit and one “intermediate-level processing” unit. In the “low-level processing” unit, we assign a location label that corresponds to a room. Then, we extract useful features from the raw sensor data. Some sensors, such as motion and door sensors, generate discrete-valued messages indicating their states (e.g., on or off, open or closed). These sensors usually only generate events when there is a change in their state, typically as a result of the direct interaction with the occupant. The discrete features used in the feature vector describe the time and the location where the events occur in the home.

**Figure 2:**
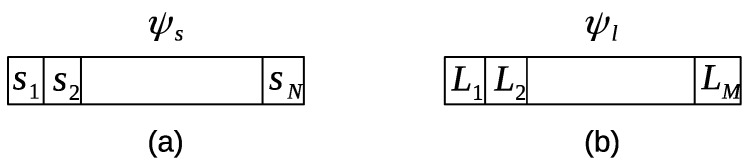
Illustration of the time-based windows. The sensor time-based window is of length $\psi_{s}$, and the time duration is computed for each sensor. The location time-based window is of length $\psi_{l}$, and the time duration is computed for each location: (**a**) sensor time-based window; (**b**) location time-based window.

In the “intermediate-level processing” step, we use a time-based windowing approach with a fixed time interval length. First, we use a small time interval length to extract the time duration within the detection area of each sensor. We will refer to it as a sensor time-based window. Each index inside the window represents a particular sensor that is installed in an environment. The value of each position in the window is the time duration of a particular sensor. The time duration in a sensor time-based window is defined as the amount of time a person spends around the detection area of a sensor. Figure 2a shows an example of a sensor time-based window. Each window has a fixed number of entries *N*, where *N* is the number of the sensors installed in an environment, and $\psi_{s}$ is the time interval length. Each entry in the window has a time duration that a person spent within the detection area of a sensor.

Secondly, we use a large time interval length to extract the time duration within the detection of each location. We will refer to it as a location time-based window. Each index inside the window represents a particular location that exists in an environment. The value of each position in the window is the time duration of a particular location. The time duration in a location time-based window is defined as the amount of time a person spent around the detection area of a location. Figure 2b shows an example of a location time-based window. Each window has a fixed number of entries *M*, where *M* is the number of the locations in an environment, and $\psi_{l}$ is the time interval length. Each entry in the window has a time duration that a person spent in a location.

The high-level processing component includes three discovery steps; namely, “intra-day pattern discovery”, “inter-day pattern discovery”, and “clustering refinement”. In the “intra-day pattern discovery” step, we want to cluster similar time intervals within the same day, where each group of similar time intervals will represent a particular pattern. First, we compare the location time-based windows. Two location time-based windows are considered similar if the overall time duration difference between the locations is small (e.g., zero or less than a certain threshold). According to this criterion, the windows are clustered accordingly. Next, a mapping step is performed in order to group the sensor time-based windows according to the clusters of the location time-based windows.

Furthermore, the sensor time-based windows are clustered, so that we can compare the durations of all sensors. Using the same criterion, two sensor time-based windows are considered similar if the overall time duration difference between the sensors is small (e.g., zero or less than a certain threshold). Finally, the sensor time-based windows are clustered within a day. Each cluster will contain a group of sensor time-based windows, where all the windows share similar time durations. The similar group of windows will represent a specific pattern of behavior (e.g., sleeping, eating). Finally, a single day is segmented into several clusters, where each cluster represents a group of sensor time-based windows. We will refer to a cluster here as a pattern.

There could be similar patterns between two days or more. The discovered patterns are unique within a day but they are not unique between days. The “inter-day pattern discovery” step aims at finding the common patterns between two or more days and clusters them, and retains only the general frequent patterns and their variations across all days. We use the Earth mover’s distance (EMD) as a similarity measure for evaluating the patterns between days. In order to provide more compressed and defined characterizations of the common frequent patterns across all days, we apply an aggregate hierarchical clustering algorithm in the third step. Finally in the last component, the clusters are then used to track and recognize the occupant’s activities.

## 4. Low-Level Processing

**Figure 3:**
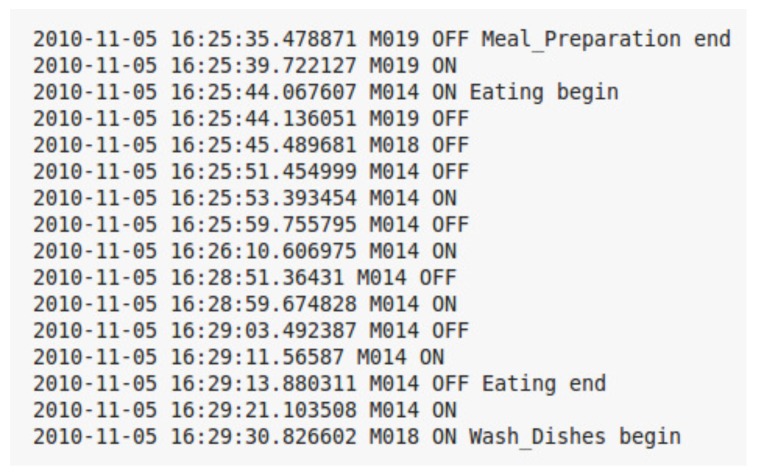
Sample raw and activity annotated sensor data. Sensors’ IDs starting with M represent motion sensors while IDs starting with D represent door sensors [32].

The data sets used in this work consist of a series of PIR motion sensor activation events. They represent times in which an occupant or a guest moved within the detection area of one of the PIRs used in the deployment. Figure 3 shows an example of several labeled sensor readings from one of the data sets. Each sensor event is described by several elements, such as a date, time of the occurrence, sensor identifier, sensor status, and associated activity label. Sensors’ identifiers starting with M represent motion sensors, and those starting with D represent door sensors. Some of the events are part of a labeled activity and others have no activity labels. The goal of an activity recognition algorithm is to predict the label of a sensor event or a sequence of sensor events.

**Figure 4:**
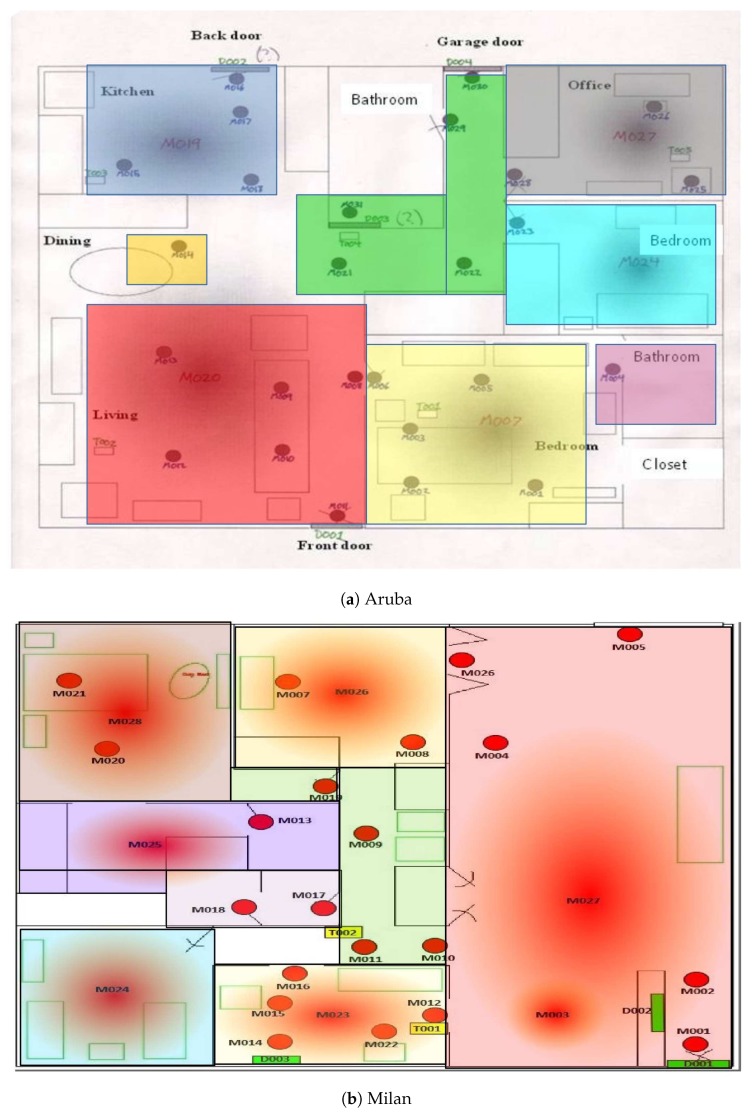
Sensor layout and location maps of apartment 1 and apartment 2. (**a**) Apartment 1 with location overlay [60]; (**b**) apartment 2 with location overlay [32].

As a first step in forming the time-based features that we will later use for the pattern discovery, we assign to each sensor event a label that corresponds to a location where the occupant is currently located. The location labeling approach was used previously in [5,57,58,59] to hypothesize that activities in a home are closely related to specific locations of a home; for example, cooking mostly occurs in the kitchen, and sleeping occurs mostly in the bedroom. Figure 4 shows the different locations as different colored boxes overlain on the deployment map for apartment 1 and apartment 2. The two apartments are code-named “Milan” [32] and “Aruba” [60]. The location labels include: kitchen, dining area, living room, bedroom, guest bedroom, office, hallway, and outside. Table 1 lists the location identifiers used. The outside of apartment is extracted using opened/closed door sensor events. Our approach of detecting outside of apartment will be explained in Section 5.2.

**Figure 5:**
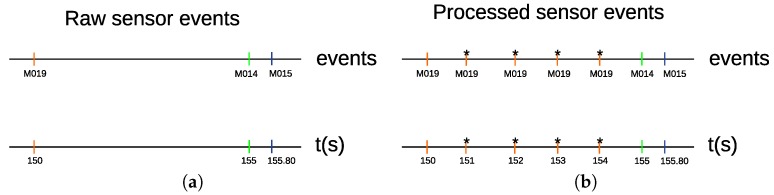
Illustration of the processing of the raw sensor events. The various binary sensor events are identified by the colored straight upward lines. The color shows the kind and the position of the sensor that was activated. The sensor data are processed to be sampled at a constant time interval of 1 s. The “*” sign indicates a sampled sensor event at a time interval of 1 s: (**a**) raw sensor events; (**b**) processed sensor events.

In “Milan” and “Aruba” data sets, the state of the sensor data is not sampled at a constant time interval. A sensor reports an on state, when there is a motion within its detection area. Otherwise, it reports an off state. The sensors were not configured to report their states at constant time intervals. Our method relies on a time-based segmentation approach. In this approach, it is essential for every time interval to have a constant amount of time duration data. In Figure 5a, there are two sensors, “M019” and “M014”. The “M019” sensor triggers an event at time “150”, and the “M014” sensor triggers an event at time “155”. The “M019” sensor does not report its state at a constant time interval till “M014” triggers an event. Therefore, we need to process the time interval between “M019” and “M014”. In order to do that, we sample the interval between “M019” and “M014” at a constant time of 1 s. Figure 5b shows the processed time interval between “M019” at time 150 and “M014” at time 155, where four “M019” events are sampled at a time interval of 1 s. The “*” sign indicates that a sensor event is sampled at a time interval of 1 s. The sensor data in “Milan” and “Aruba” data sets are processed to be sampled at a constant time interval of 1 s. Special care has to be taken for edge cases when two sensor events occur at times that are less than 1 s. For such a case, no sampling is performed. For instance, the time interval between “M014” at time 155, and “M015” at 155.80 was not sampled, as shown in Figure 5b.

Given a sampled event at a constant time interval ${\vec{e}}_{i}=\left(\eta_{i},s_{i},l_{i},d_{i}\right)$ where $\eta_{i}$ denotes the timestamp in milliseconds, $s_{i}$ denotes the sensor ID, $l_{i}$ denotes the location ID, and $d_{i}$ denotes the duration in milliseconds between two sensor events, and it is computed as follows:

(1) $$d_{i}=t_{i+1}-t_{i}$$

**Figure 6:**
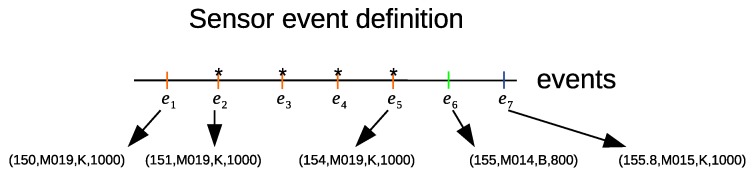
Illustration of the sensor event definition. Each sensor event contains four pieces of information: timestamp, sensor ID, location, and duration.

We define a day instance as a sequence of *r* sampled sensor events $\left({\vec{e}}_{1},{\vec{e}}_{2},\ldots {\vec{e}}_{r}\right)$. Figure 6 shows examples of sensor event definitions for several sensor event outputs. The first five sensor events are activated in the kitchen, followed by the activation of the sensor events in the bathroom. It can be noted that these sensor events are sampled at a constant time interval of 1 s.

## 5. Intermediate-Level Processing

### 5.1. Feature Extraction

We use a time-based windowing approach to divide the entire sequence of the sampled sensor events into equal size time intervals. It is challenging to choose the ideal length of time interval. An interval that is too small will incorporate noise of the signal, while an interval that is too large will smooth out important details of the signal. Therefore, we use two time interval lengths. The first time interval length is small in order to capture the movement of a human being. We would like the discretization of such movement to correspond closely to the action primitives that are performed. Discretizing with a small interval might pick up any relevant activity information given the small pauses in the movement of a human being around the detection areas of the sensors. On the other hand, discretizing with a large interval length might capture long-term activity, such as relaxation or sleep that contains large pauses in the movement of a human being around the detection areas of the locations.

In the case of activity discovery using motion sensor data, no existing work presents any empirical or theoretical standing to select any time interval length. Some preprocessing steps are often employed in order to convert the raw time series data into a different representation, which makes the discovery of the patterns easier. For motion sensor data, there is no known conversion that provides better results in activity discovery, than using the raw sensor data. We define a time interval as grouping the sensor events into intervals of seconds, minutes, and hours. A longer length of time can be divided into a number of shorter periods of time, all of the same length.

We define two different time intervals lengths $\psi_{s}$ and $\psi_{l}$, such that $\psi_{s}\leq \psi_{l}$. The time interval length $\psi_{s}$ is referred to a sensor time interval length, and this is indicated by the subscript *s* in the symbol $\psi_{s}$. The time interval length $\psi_{l}$ is referred to a location time interval length, and this is indicated by the subscript *l* in the symbol $\psi_{l}$. The subscripts *s* and *l* will be used in our notation to indicate whether a feature vector belongs to a sensor or a location time interval.

Formally, the sequence of the sampled sensor events $\left({\vec{e}}_{1},{\vec{e}}_{2},\ldots {\vec{e}}_{r}\right)$ is divided using $\psi_{s}$ into windows of equal time intervals $\left(\Delta_{1}^{s},\Delta_{2}^{s},\ldots \Delta_{n}^{s}\right)$, and the $\Delta_{p}^{s}$ window is represented by the sequence $\left[{\vec{e}}_{j-\psi_{s}},{\vec{e}}_{j}\right]$. The value of the variable $\psi_{s}$ changes based on the experimental setting. It is obtained through an experiential procedure by examining the impacts of the various outcomes of $\psi_{s}$ on the performance of the classification system. The exact value of $\psi_{s}$ will be discussed in Section 8.

Once the sensor time interval $\Delta_{p}^{s}$ is defined, the next step is to change this time interval into a feature vector that encapsulates its statistical figures. We execute this step by forming a constant length feature vector ${\vec{f}}_{p}^{s}$, explicitly encapsulating the time durations of the different sensors within the time interval. With *N* sensors installed in a smart home, the dimension of the vector ${\vec{f}}_{p}^{s}$ will be *N*. A time duration is defined as the amount of time a person spends around the detection area of a sensor or a location. The sum of all time durations serves as a signal of how much motion and information flow are happening (or not happening) around the detection area of a sensor or a location within a time interval.

**Figure 7:**
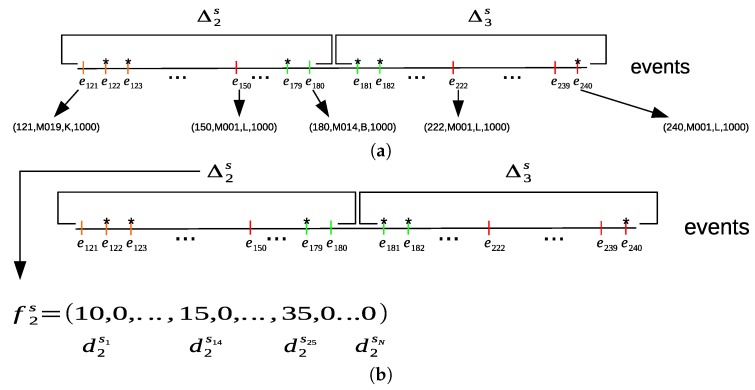
Illustration of dividing the sequence of the sensor events using a sensor time interval $\psi_{s}$ in order to form the sensor feature vectors: (**a**) sensor time interval; (**b**) sensor feature vector.

Let $d_{p}^{s_{\hat{u}}}$ be the sum of all time durations for a sensor $s_{\hat{u}}$ in ${\vec{f}}_{p}^{s}$ from a time interval $\Delta_{p}^{s}$, and it can be computed as follows:

(2) $$d_{p}^{s_{\hat{u}}}=\sum_{{\vec{e}}_{j}\in C^{s}\left(\Delta_{p}^{s},s_{\hat{u}}\right)}d_{j}$$

where $C^{s}\left(\Delta_{p}^{s},s_{\hat{u}}\right)=\left\{{\vec{e}}_{j}=\left(\eta_{j},s_{j},l_{j},d_{j}\right):\left({\vec{e}}_{j}\in \Delta_{p}^{s}\right)\wedge \left(s_{j}=s_{\hat{u}}\right)\right\}$. Finally, for each sensor time interval $\Delta_{p}^{s}$, we will have a corresponding sensor feature vector ${\vec{f}}_{p}^{s}=\left(d_{p}^{s_{1}},d_{p}^{s_{2}},\ldots d_{p}^{s_{N}}\right)$, where the sum of all time duration for each sensor is computed within a time interval $\Delta_{p}^{s}$. Figure 7a shows an example of dividing the sequence of the sampled sensor events into sensor time intervals. For each sensor time interval, the sum of all time durations of each sensor is computed from the sensor events within its sensor time interval to construct a sensor feature vector ${\vec{f}}_{p}^{s}$, as shown in Figure 7b.

Similarly, we use the location time interval length to divide the sequence of the sampled sensor events $\left({\vec{e}}_{1},{\vec{e}}_{2},\ldots {\vec{e}}_{r}\right)$ into windows of an equal time intervals $\left(\Delta_{1}^{l},\Delta_{2}^{l},\ldots \Delta_{m}^{l}\right)$, and the $\Delta_{q}^{l}$ window is represented by the sequence $\left[{\vec{e}}_{j-\psi_{l}},{\vec{e}}_{j}\right]$. The value of the variable $\psi_{l}$ changes based on the experimental setting. It is obtained through an experiential procedure by examining the impacts of the various outcomes of $\psi_{l}$ on the performance of the classification system. The exact value of $\psi_{l}$ will be discussed in Section 8.

**Figure 8:**
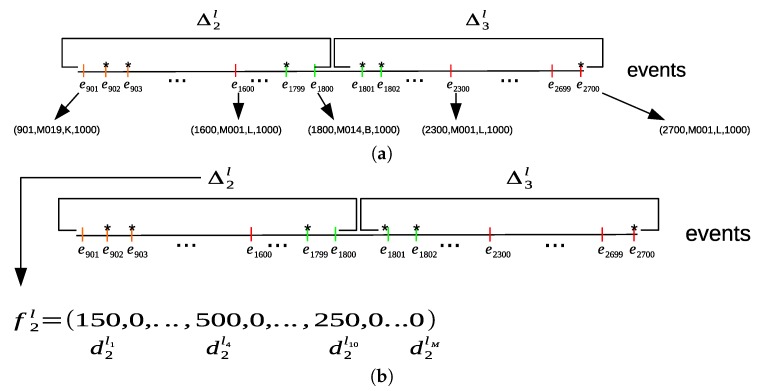
Illustration of dividing the sequence of the sensor events using a location time interval $\psi_{l}$ in order to form location feature vectors: (**a**) location time interval; (**b**) location feature vector.

Once the location time interval $\Delta_{q}^{l}$ is defined, the next step is to change this time interval into a feature vector that encapsulates its statistical figures. We execute this step by forming a constant length feature vector ${\vec{f}}_{q}^{l}$, explicitly encapsulating the time durations of the different locations within the time interval. With *M* locations in a smart home, the dimension of the vector ${\vec{f}}_{q}^{l}$ will be *M*. Let $d_{q}^{l_{\hat{e}}}$ be the sum of all time durations for location $l_{\hat{e}}$ in ${\vec{f}}_{q}^{l}$ from a time interval $\Delta_{q}^{l}$, and it can be computed as follows:

(3) $$d_{q}^{l_{\hat{e}}}=\sum_{{\vec{e}}_{j}\in C^{l}\left(\Delta_{q}^{l},l_{\hat{e}}\right)}d_{j}$$

where $C^{l}\left(\Delta_{q}^{l},l_{\hat{e}}\right)=\left\{{\vec{e}}_{j}=\left(\eta_{j},s_{j},l_{j},d_{j}\right):\left({\vec{e}}_{j}\in \Delta_{q}^{l}\right)\wedge \left(l_{j}=l_{\hat{e}}\right)\right\}$. Finally, for each location time interval $\Delta_{q}^{l}$, we will have a corresponding location feature vector ${\vec{f}}_{q}^{l}=\left(d_{q}^{l_{1}},d_{q}^{l_{2}},\ldots d_{q}^{l_{M}}\right)$, where the sum of all time durations for each location is computed within a time interval $\Delta_{q}^{l}$. Figure 8a shows an example of dividing the sequence of the sampled sensor events into location time intervals. For each location time interval, the sum of all time durations of each location is computed from the sensor events within its location time interval to construct a location feature vector ${\vec{f}}_{q}^{l}$, as shown in Figure 8b.

Our objective in the following sections is to use the sensor feature vectors and the location feature vectors to discover the activity patterns. We introduce three levels of clustering; i.e., intra-day clustering in order to discover patterns within the same day, inter-day clustering in order to discover more general frequent patterns between days, and finally, aggregate hierarchical clustering is used to provide a more compact and precise representation of the discovered activity patterns. In the following section, we extend the definition of the sensor feature vector and the location feature vector by adding two additional feature values that represent whether an entrance to the home or exit from the home occurred.

### 5.2. Outside Apartment Feature Representation

In order to avoid sensor events that are far apart in time, we resorted to sampling the sensor events at a constant time interval of 1 s. As a result of the sampling, labels were assigned to all locations in the smart homes. The exit doors are located in different places in the home. There could be exit doors in the hallway, or in the kitchen, as shown in Figure 8. Therefore, a location label is assigned to the door when it is opened or closed according to its place in the smart home.

It is important to distinguish between two activities: LEAVE_HOME and ENTER_HOME. Using the door sensor events to detect the times when a user leaves or enters a home did not yield accurate results. The door sensors may not report their state correctly and accurately when the door is opened or closed. During the closing and opening of the door multiple times, many motion sensor events could be triggered and mixed with the door sensor events in the same window, and this makes it difficult to distinguish between the two activities using a specific order of the triggered sensors in the window. Additionally, the start and the end label markers sometimes are not set correctly to the sensor event of interest (e.g., door sensor events).

**Figure 9:**
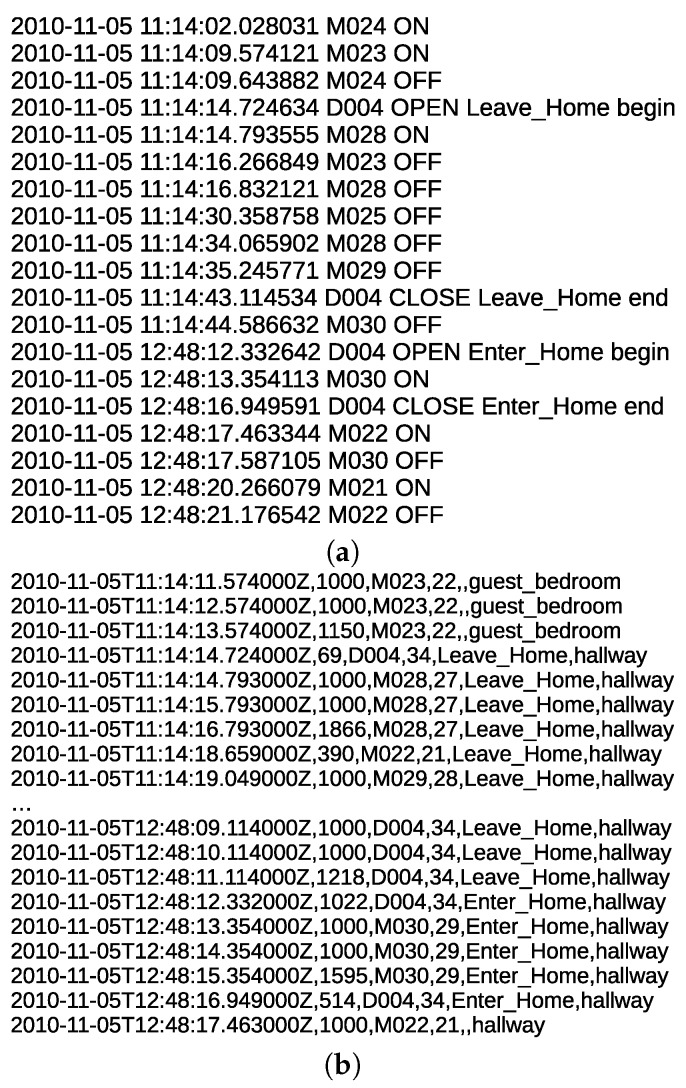
Illustration of the sensor events for LEAVE_HOME and ENTER_HOME activities. There is a gap of one and a half hours. The sensor events are sampled at a constant time interval of 1 s. A location label is assigned to each sampled sensor event: (**a**) sensor events before sampling; (**b**) sensor events after sampling.

We need to extend our feature vector definition to indicate when a user leaves and enters a home. Figure 9a shows an example of the door sensor events before sampling the events. There is a gap of one and a half hours without any sensor events being reported. Figure 9b shows an example of the door sensor events after sampling the events. The sensor events are reported periodically at a constant time interval of 1 s.

We extend the sensor feature vector ${\vec{f}}_{p}^{s}$ by defining two additional feature values. The dimension of the vector ${\vec{f}}_{p}^{s}$ will be N+2. Let $d_{p}^{s_{N+1}}$ be the N+1 feature value, and it will be the feature value that represents the ENTER_HOME activity. Let $d_{p}^{s_{N+2}}$ be the N+2 feature value, and it will be the feature value that represents the LEAVE_HOME activity. The default values of $d_{p}^{s_{N+1}}$ and $d_{p}^{s_{N+2}}$ are 0. This means the user did not perform any ENTER_HOME or LEAVE_HOME activities during a time interval $\Delta_{p}^{s}$. Similarly, we extend the location feature vector ${\vec{f}}_{q}^{l}$ by defining two additional feature values. The dimension of the vector ${\vec{f}}_{q}^{l}$ will be M+2. Let $d_{q}^{l_{M+1}}$ be the M+1 feature value, and it will be the feature value that represents the ENTER_HOME activity. Let $d_{q}^{l_{M+2}}$ be the M+2 feature value, and it will be the feature value that represents the LEAVE_HOME activity. The default values of $d_{q}^{l_{M+1}}$ and $d_{q}^{l_{M+2}}$ are 0. This means the user did not perform any ENTER_HOME or LEAVE_HOME activities during a time interval $\Delta_{q}^{l}$.

**Figure 10:**
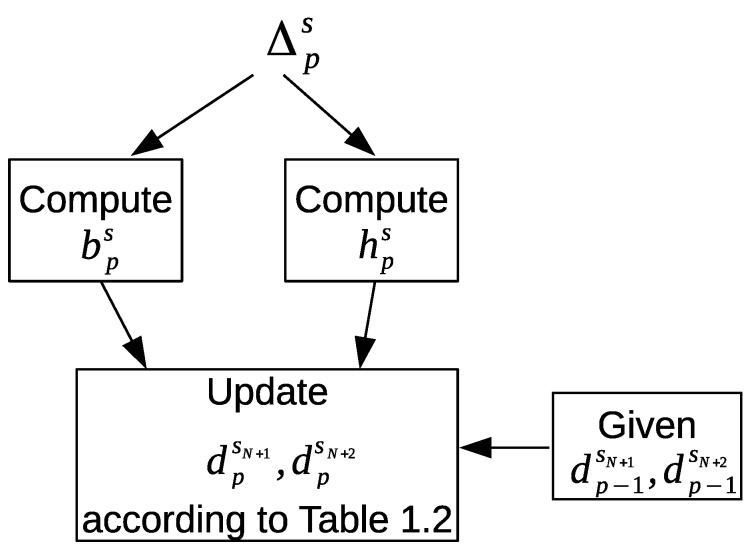
Illustration of our approach for updating the sensor features values of $d_{p}^{s_{N+1}}$ and $d_{p}^{s_{N+2}}$ in order to represent the ENTER_HOME and LEAVE_HOME activities.

Figure 10 shows our proposed approach to update the newly introduced feature values that will represent the LEAVE_HOME and ENTER_HOME activities. First, the frequency of the motion sensor event counts $b_{p}^{s}$ is computed for a time interval $\Delta_{p}^{s}$ as follows:

(4) $$b_{p}^{s}=\frac{\sum_{{\vec{e}}_{j}\in D^{b}\left(\Delta_{p}^{s}\right)}1}{|\Delta_{p}^{s}|}$$

where $D^{b}\left(\Delta_{p}^{s}\right)=\left\{{\vec{e}}_{j}=\left(\eta_{j},s_{j},l_{j},d_{j}\right):\left({\vec{e}}_{j}\in \Delta_{p}^{s}\right)\wedge \left(s_{j}\mathrm{is}\;\mathrm{motion}\;\mathrm{sensor}\right)\right\}$. Similarly, the frequency of the door sensor event counts $h_{p}^{s}$ for a time interval $\Delta_{p}^{s}$ is computed as follows:

(5) $$h_{p}^{s}=\frac{\sum_{{\vec{e}}_{j}\in D^{h}\left(\Delta_{p}^{s}\right)}1}{|\Delta_{p}^{s}|}$$

where $D^{h}\left(\Delta_{p}^{s}\right)=\left\{{\vec{e}}_{j}=\left(\eta_{j},s_{j},l_{j},d_{j}\right):\left({\vec{e}}_{j}\in \Delta_{p}^{s}\right)\wedge \left(s_{j}\mathrm{is}\;\mathrm{door}\;\mathrm{sensor}\right)\right\}$. Next, the values of $d_{p}^{s_{N+1}}$ and $d_{p}^{s_{N+2}}$ are updated according to the defined conditions in Table 2 to indicate whether a sensor feature vector ${\vec{f}}_{p}^{s}$ represents the ENTER_HOME or LEAVE_HOME activities. Each condition in Table 2 depends on the following information:

- The values of $d_{p-1}^{s_{N+1}}$ and $d_{p-1}^{s_{N+2}}$ from the previous time interval $\Delta_{p-1}^{s}$.
- The frequency of the motion sensor events $b_{p}^{s}$, and the frequency of the door sensor events $h_{p}^{s}$ from the current time interval $\Delta_{p}^{s}$.

**Figure 11:**
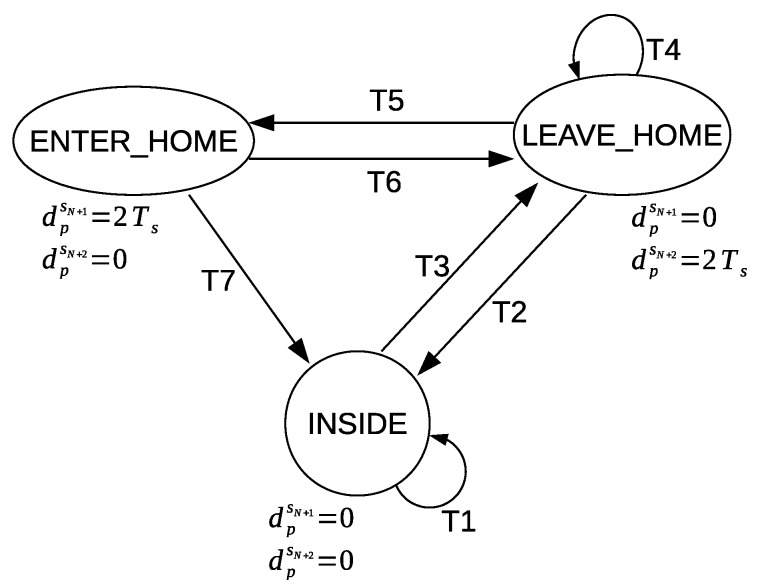
A graphical representation of the state transitions to extend the sensor feature values in order to represent the ENTER_HOME and LEAVE_HOME activities.

The transitions in the graphical representation of Figure 11 are based on the defined conditions in Table 2. The nodes represent the ENTER_HOME and LEAVE_HOME activities. The third node represents the times of no detection of the ENTER_HOME and LEAVE_HOME activities; we will refer to this node as NO_LEAVE_NO_ENTER (inside home). The values given to $d_{p}^{s_{N+1}}$ and $d_{p}^{s_{N+2}}$ are shown below each node. The default values of $d_{p}^{s_{N+1}}$ and $d_{p}^{s_{N+2}}$ are always zero unless one of the defined conditions is met; then they are updated accordingly to the given values in order to represent a corresponding ENTER_HOME activity or LEAVE_HOME activity for a sensor feature vector ${\vec{f}}_{p}^{s}$.

When a sensor feature vector ${\vec{f}}_{p}^{s}$ contains zero values for $d_{p}^{s_{N+1}}$ and $d_{p}^{s_{N+2}}$, that means this feature vector represents a different activity than ENTER_HOME or LEAVE_HOME. A value of $2\psi_{s}$ is assigned to $d_{p}^{s_{N+1}}$, and a value of zero is assigned to $d_{p}^{s_{N+2}}$ in order to represent ENTER_HOME activity. On the other hand, a value of zero is assigned to $d_{p}^{s_{N+1}}$, and a value of $2\psi_{s}$ is assigned to $d_{p}^{s_{N+2}}$ in order to represent LEAVE_HOME activity.

A location feature vector ${\vec{f}}_{q}^{l}$ will represent the ENTER_HOME activity or LEAVE_HOME activity by following the same approach to compute the values of $d_{q}^{l_{M+1}}$ and $d_{q}^{l_{M+2}}$ as in the sensor feature vector case.

## 6. High-Level Processing

### 6.1. Intra-Day Pattern Discovery

As previously mentioned, with a fixed length time interval over the sensor events, it is possible for two sensors to be in two different time intervals, but both of them have the same time duration. In order to group similar time intervals together into clusters, where each cluster will represent a specific pattern, we measure the similarity between the different sensors’ time intervals and the locations time intervals using the duration information encoded in their time intervals. For that, we use an exponential function to compute the similarity between the time intervals.

We define ${\vec{F}}_{k}^{s}=\left({\vec{f}}_{1}^{s},{\vec{f}}_{2}^{s},\ldots {\vec{f}}_{n}^{s}\right)$ to represent all the sensor feature vectors for a day *k*, where *n* is the number of the sensor feature vectors in a day *k*. We define ${\vec{c}}_{k}^{s}=\left(o_{1}^{s},o_{2}^{s},\ldots o_{n}^{s}\right)$ to be the cluster IDs that are assigned to each sensor feature vector in ${\vec{F}}_{k}^{s}$ for a day *k*. We will refer to them as the sensor clusters. Similarly, we define ${\vec{F}}_{k}^{l}=\left({\vec{f}}_{1}^{l},{\vec{f}}_{2}^{l},\ldots {\vec{f}}_{m}^{l}\right)$ to represent all the location feature vectors for a day *k*, where *m* is the number of location feature vectors in a day *k*. We define ${\vec{c}}_{k}^{l}=\left(o_{1}^{l},o_{2}^{l},\ldots o_{m}^{l}\right)$ to be the cluster IDs that are assigned to each location feature vector in ${\vec{F}}_{k}^{l}$ for a day *k*. We will refer to them as the location clusters.

**Figure 12:**
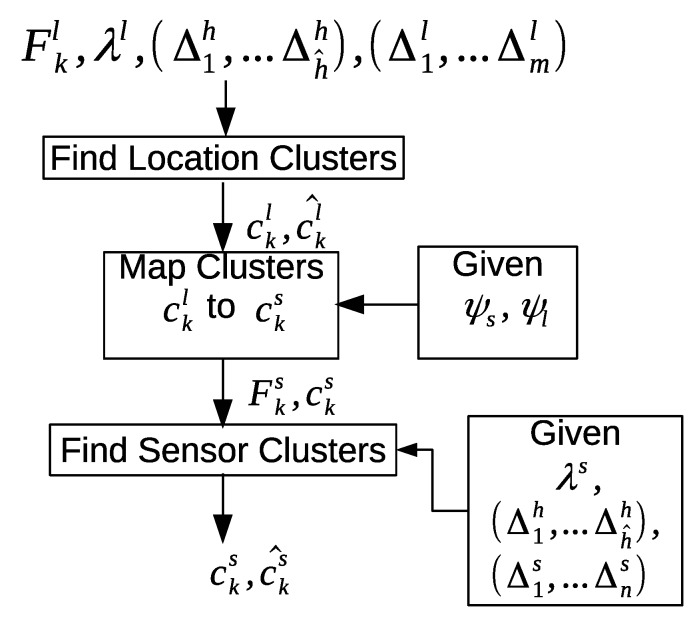
An overview of the intra-day discovery patterns algorithm.

Figure 12 shows an overview of the intra-day pattern discovery approach. First, we find the location clusters ${\vec{c}}_{k}^{l}$ for the location feature vectors ${\vec{F}}_{k}^{l}$. Next, we assign the location cluster IDs ${\vec{c}}_{k}^{l}$ to the sensor cluster IDs ${\vec{c}}_{k}^{s}$. Finally, we find the sensor clusters ${\vec{c}}_{k}^{s}$ for the sensor feature vectors ${\vec{F}}_{k}^{s}$.

#### 6.1.1. Finding the Location Clusters

Humans and animals are living beings; their lives are centered around habits. As a consequence, many of our daily routines center around certain hours. For instance, occupants normally go to bed at night, are active in the morning, and consume food at evenly fixed time intervals during their waking hours. Because users have basically the same kinds of routines, the concept of a clock let us to harmonize group routines, such as gatherings and physical activity events around the clock, week, month, and year units such as an hour in a day, a day in a week, a week in a month, and a month in a year. Let $\psi_{h}$ be an hourly time interval length (e.g., from 14:00 to 15:00), where $\psi_{s}<\psi_{l}<\psi_{h}$. We use the hourly time interval length $\psi_{h}$ to divide the sequence of sampled sensor events into windows of an equal time intervals $\left(\Delta_{1}^{h},\Delta_{2}^{h},\ldots \Delta_{\hat{h}}^{h}\right)$, where $\hat{h}$ is the number of the hourly time intervals, and the $\Delta_{a}^{h}$ window is represented by the sequence $\left[e_{j-\psi_{h}},e_{j}\right]$.

We use an exponential decay model to measure the similarity between the two location feature vectors as follows:

(6) $$Y^{l}\left(i,j\right)=\exp^{\left(-\lambda^{l}{\hat{y}}^{l}\left(i,j\right)\right)}$$

where ${\hat{y}}^{l}\left(i,j\right)$ is computed as follows:

(7) $${\hat{y}}^{l}\left(i,j\right)=\sum_{\begin{array}{c} c=1\\ \Delta_{i}^{l}\subseteq \Delta_{a}^{h}\\ \Delta_{j}^{l}\subseteq \Delta_{a}^{h} \end{array}}^{M+2}|d_{i}^{l_{c}}-d_{j}^{l_{c}}|$$

**Figure 13:**
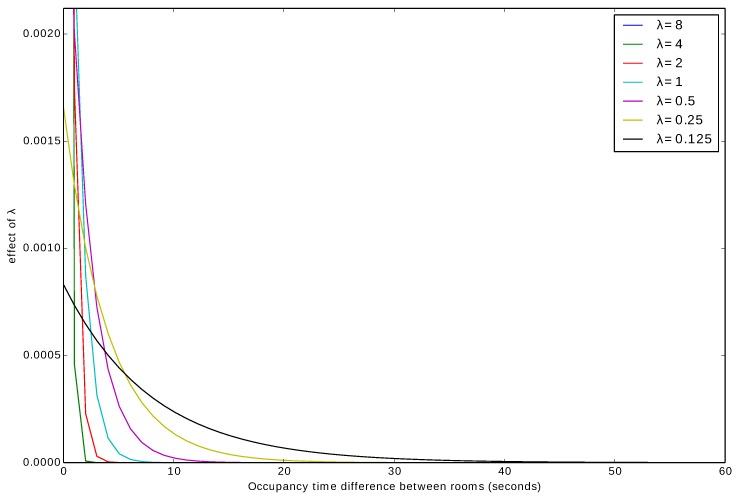
Effect of λ on the degree of the similarity.

The *i*th time interval $\Delta_{i}^{l}$ and the *j*th time interval $\Delta_{j}^{l}$ should be part of the hourly time interval $\Delta_{a}^{h}$ in order to group the feature vectors around specific times. The value of $\lambda^{l}$ determines the degree of the similarity between the two location feature vectors. Figure 13 shows an illustration of the parameter λ’s effect on the degree of the similarity. If λ>1, then only the time duration differences in the *i*th feature vector are temporally very close to the time duration differences in the *j*th feature vector. With 0<λ<1, the degree of the similarity allows for a wider range of the time duration difference. When λ=0, the degree of the similarity has no influence, and the time duration differences in both feature vectors are identical.

**Figure 14:**
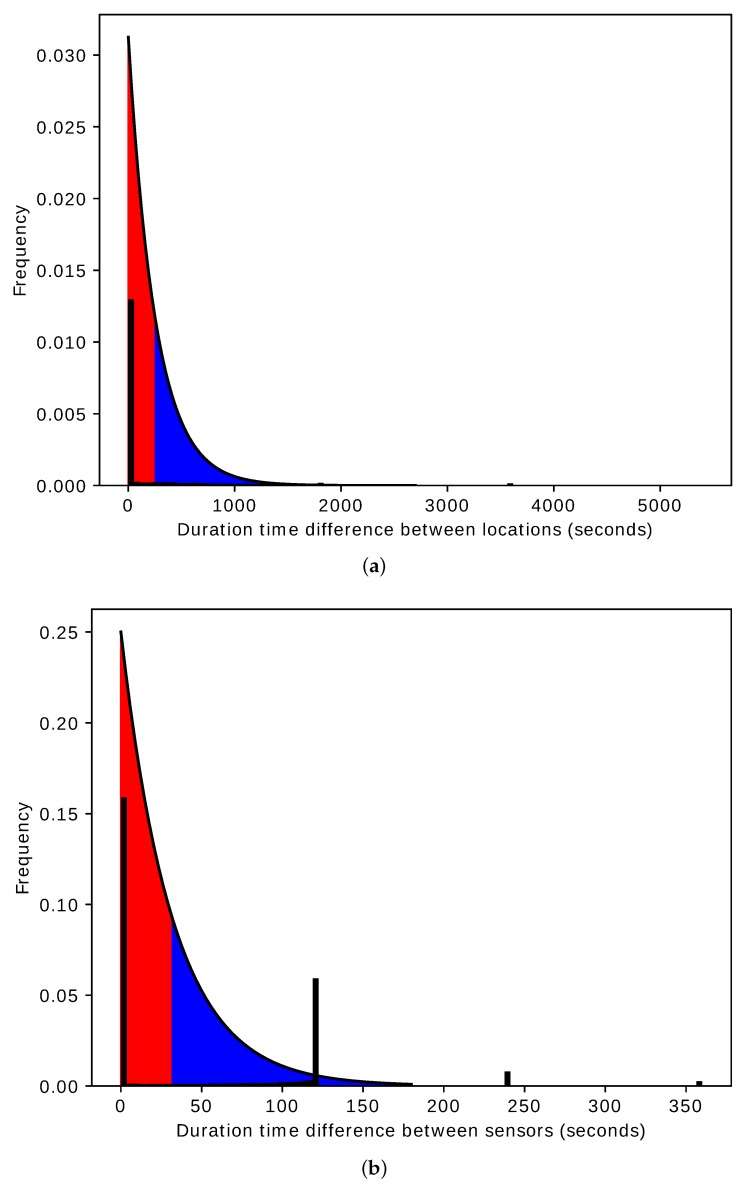
The exponential decay model dentition. The red shaded area represents the first half of the area under the curve, in which two feature vectors are considered similar, while the blue shaded represents the second half of the area under the curve, in which two feature vectors are considered dissimilar: (**a**) exponential decay model for the location feature vectors with $\psi_{l}=900$ s and $\lambda^{l}=2^{-8}$; (**b**) exponential decay model for sensor feature vectors with $\psi_{s}=60$ s and $\lambda^{s}=2^{-5}$.

Algorithm 1 shows how the location feature vectors are grouped into clusters. First ${\vec{c}}_{k}^{l}$ is initialized to zero; that means all the location feature vectors belong to a single cluster (same cluster). The similarity between each pair of the location feature vectors is computed using Equation (6). The similarity value is evaluated to determine whether the two location feature vectors belong to the same cluster or not. We use the area under the curve of the exponential decay model as an evaluation criterion. Figure 14a shows an exponential decay model with $\lambda^{l}=2^{-8}$. When the similarity value $Y^{l}\left(i,j\right)$ falls within the first half of the area under the curve—$1-Y^{l}\left(i,j\right)<0.5$ (red shaded area), then the two location feature vectors are considered similar, and assigned to the same cluster. Otherwise, they do not belong to the same cluster (blue shaded area).
**Algorithm 1** findLocationClusters.   **Input:**
${\vec{F}}_{k}^{l},\lambda^{l},\left(\Delta_{1}^{h},\Delta_{2}^{h},\ldots \Delta_{\hat{h}}^{h}\right),\left(\Delta_{1}^{l},\Delta_{2}^{l},\ldots \Delta_{m}^{l}\right)$      **Output: ${\vec{c}}_{k}^{l},{\hat{\vec{c}}}_{k}^{l}$**1:${\vec{c}}_{k}^{l}\leftarrow \vec{0}$2:clusterID=13:${\hat{o}}_{clusterID}^{l}=clusterID$4:**for**i←1 to *m* **do**5:  updateCluster=false6:  **if** $o_{i}^{l}=0$ **then**7:    **for** j←1 to *m* **do**8:      **for** a←1 to $\hat{h}$ **do**9:        compute $Y^{l}\left(i,j\right)$ as in Equation (6)10:        **if** $\left(1-Y^{l}\left(i,j\right)<0.5\wedge o_{j}^{l}=0\right)$ **then**11:          $o_{j}^{l}=clusterID$ //assign *i* and *j* to the same cluster12:          updateCluster=true13:        **end if**14:      **end for**15:    **end for**16:    **if** (updateCluster) **then**17:      clusterID=clusterID+118:      ${\hat{o}}_{clusterID}^{l}=clusterID$19:    **end if**20:  **end if**21:**end for**

We define ${\hat{\vec{c}}}_{k}^{l}=\left({\hat{o}}_{1}^{l},{\hat{o}}_{2}^{l},\ldots {\hat{o}}_{\hat{m}}^{l}\right)$ to be the set of distinct location cluster IDs in ${\vec{c}}_{k}^{l}$; then $\hat{m}$ is the number of distinct location cluster IDs occurring in ${\vec{c}}_{k}^{l}$. The location clusters contain mainly a small number of clusters. These clusters represent the long-term frequent patterns, such as relaxing, sleeping, and working that do not require active movements between the locations.

#### 6.1.2. Mapping Clusters

After grouping the location feature vectors ${\vec{F}}_{k}^{l}$ to their corresponding location clusters ${\vec{c}}_{k}^{l}$, we initially group the sensor feature vectors ${\vec{F}}_{k}^{s}$ into clusters using the location cluster IDs ${\vec{c}}_{k}^{l}$. This is done by assigning the location cluster IDs ${\vec{c}}_{k}^{l}$ to the sensor cluster IDs ${\vec{c}}_{k}^{s}$, as shown in Algorithm 2. The cluster mapping between ${\vec{c}}_{k}^{l}$ and ${\vec{c}}_{k}^{s}$ is subject to the following constraint:

(8) $$\psi_{l}\;\mathrm{mod}\;\psi_{s}\equiv 0$$

**Algorithm 2** mapClusters.   **Input:**
${\vec{c}}_{k}^{l},{\hat{\vec{c}}}_{k}^{l},\psi_{s},\psi_{l}$      **Output: ${\vec{c}}_{k}^{s}$**1:${\vec{c}}_{k}^{s}\leftarrow \vec{0}$2:**for**i←1 to $\hat{m}$ **do**3:  **for** j←1 to *m* **do**4:    **if** ${\hat{o}}_{i}^{l}=o_{j}^{l}$ **then**5:      $x=\frac{\psi_{l}\cdot j}{\psi_{s}}$6:      $y=\frac{\psi_{l}\cdot \left(j+1\right)}{\psi_{s}}$7:      **for** x←1 to *y* **do**8:        $o_{x}^{s}={\hat{o}}_{i}^{l}$9:      **end for**10:    **end if**11:  **end for**12:**end for**

In the following section, we will evaluate the similarity between the sensor feature vectors in order to group the similar sensor feature vectors into clusters, where each cluster will represent a specific pattern.

#### 6.1.3. Finding the Sensor Clusters

Similarly to finding the location clusters, we use an exponential decay model to measure the similarity between two sensor feature vectors as follows:

(9) $$Y^{s}\left(i,j\right)=\exp^{\left(-\lambda^{s}{\hat{y}}^{s}\left(i,j\right)\right)}$$

where ${\hat{y}}^{s}\left(i,j\right)$ is computed as follows:

(10) $${\hat{y}}^{s}\left(i,j\right)=\sum_{\begin{array}{c} c=1\\ \Delta_{i}^{s}\subseteq \Delta_{a}^{h}\\ \Delta_{j}^{s}\subseteq \Delta_{a}^{h} \end{array}}^{N+2}|d_{i}^{s_{c}}-d_{j}^{s_{c}}|$$

The *i*th time interval $\Delta_{i}^{s}$ and *j*th time interval $\Delta_{j}^{s}$ should be part of the hourly time interval $\Delta_{a}^{h}$ in order to group the feature vectors around specific times. The value of $\lambda^{s}$ determines the degree of the similarity between the two sensor feature vectors. Algorithm 3 shows how the sensor feature vectors are grouped into clusters. The similarity between each pair of sensor feature vectors is computed using Equation (9). The similarity value is evaluated to determine whether the two sensor feature vectors belong to the same cluster or not. We use the area under the curve of the exponential decay model as an evaluation criterion. Figure 14b shows an exponential decay model with $\lambda^{s}=2^{-5}$. When the similarity value $Y^{s}\left(i,j\right)$ falls within the first half of the area under the curve: $1-Y^{s}\left(i,j\right)<0.5$ (red shaded area), then the two sensor feature vectors are considered similar, and assigned to the same cluster. Otherwise, they do not belong to the same cluster (blue shaded area).
**Algorithm 3** findSensorClusters.   **Input:**
${\vec{F}}_{k}^{s},{\vec{c}}_{k}^{s},\lambda^{s},\left(\Delta_{1}^{h},\Delta_{2}^{h},\ldots \Delta_{\hat{h}}^{h}\right),\left(\Delta_{1}^{s},\Delta_{2}^{s},\ldots \Delta_{n}^{s}\right)$      **Output: ${\vec{c}}_{k}^{s},{\hat{\vec{c}}}_{k}^{s}$**1:$clusterID=max({\vec{c}}_{k}^{s})+1$2:${\hat{o}}_{clusterID}^{s}=clusterID$3:**for**i←1 to *n* **do**4:  updateCluster=false5:  **if** $o_{i}^{s}=0$ **then**6:    **for** j←1 to *n* **do**7:      **for** a←1 to $\hat{h}$ **do**8:        compute $Y^{s}\left(i,j\right)$ as in Equation (9)9:        **if** $\left(1-Y^{s}\left(i,j\right)<0.5\wedge o_{j}^{s}=0\right)$ **then**10:          $o_{j}^{s}=clusterID$ //assign *i* and *j* to the same cluster11:          updateCluster=true12:        **end if**13:      **end for**14:    **end for**15:    **if** (updateCluster) **then**16:      clusterID=clusterID+1
17:      ${\hat{o}}_{clusterID}^{s}=clusterID$
18:    **end if**19:  **end if**20:**end for**

In this section, we perform a clustering within the same day, where the sensor feature vectors ${\vec{F}}_{k}^{s}$ are grouped into clusters ${\vec{c}}_{k}^{s}$; each cluster represents a specific pattern in a day *k*. Let ${\hat{\vec{c}}}_{k}^{s}=\left({\hat{o}}_{1}^{s},{\hat{o}}_{2}^{s},\ldots {\hat{o}}_{\hat{n}}^{s}\right)$ be the set of distinct sensor cluster IDs in ${\vec{c}}_{k}^{s}$; then $\hat{n}$ is the number of distinct sensor cluster IDs occurring in ${\vec{c}}_{k}^{s}$. We define a pattern ${\vec{p}}_{w}^{s}=\left({\vec{f}}_{1}^{s},{\vec{f}}_{2}^{s},\ldots {\vec{f}}_{a_{w}}^{s}\right)$ to be the group of sensor feature vectors that belongs to a cluster ${\hat{o}}_{w}^{s}$, where $a_{w}$ is the number of the sensor feature vectors that belongs to a pattern ${\vec{p}}_{w}^{s}$, and $w\in \{1,2,\ldots \hat{n}\}$. In other words, ${\vec{p}}_{w}^{s}$ is the cluster data for a cluster ${\hat{o}}_{w}^{s}$. We define ${\vec{P}}_{k}^{s}=\left({\vec{p}}_{1}^{s},{\vec{p}}_{2}^{s},\ldots {\vec{p}}_{\hat{n}}^{s}\right)$ to be the cluster data for ${\hat{\vec{c}}}_{k}^{s}$ in a day *k*.

For each sensor feature vector ${\vec{f}}_{i}^{s}$, we assign a time reference $\omega_{i}^{s}$ in the following format: “h.m”, where “h” is the time in hours, and “m” is the time in minutes. We define ${\vec{t}}_{k}^{s}=\left(\omega_{1}^{s},\omega_{2}^{s},\ldots \omega_{n}^{s}\right)$ to represent all the assigned time references to the sensor feature vectors ${\vec{F}}_{k}^{s}$ for a day *k*. Algorithm 4 shows the time reference computation and assignment. Let ${\vec{u}}_{w}^{s}=\left(\omega_{1}^{s},\omega_{2}^{s},\ldots \omega_{a_{w}}^{s}\right)$ be the time references for the cluster data ${\vec{p}}_{w}^{s}$ that belong to a cluster ${\hat{o}}_{w}^{s}$. Let ${\vec{T}}_{k}^{s}=\left({\vec{u}}_{1}^{s},{\vec{u}}_{2}^{s},\ldots {\vec{u}}_{\hat{n}}^{s}\right)$ be the time references for the cluster data ${\vec{P}}_{k}^{s}$ that belong to clusters ${\hat{\vec{c}}}_{k}^{s}$ in a day *k*.
**Algorithm 4** assignTimeRef.   **Input:**
$n,\psi_{s},\psi_{h}$      **Output: ${\vec{t}}_{k}^{s}$**1:${\vec{t}}_{k}^{s}\leftarrow \vec{0}$2:$step=\frac{\psi_{h}}{\psi_{s}}$3:h=04:increment=step5:**for**i←1;i<n;i=i+step**do**6:  m=h7:  **for** j←i;j<increment∧j<n;j=j+1 **do**8:    $t_{j}^{s}=m$
9:    m=m+0.0110:  **end for**11:  increment=increment+step
11:  h=h+1
13:**end for**

Algorithm 5 summarizes the algorithms of the intra-day pattern discovery approach. In the following section, we will use the Earth mover’s distance (EMD) to perform a clustering between days. This is a pruning stage process; it intends to considerably reduce the number of the clusters discovered within the same day, and find more general patterns across all days.
**Algorithm 5** computeIntraClusters   **Input:**
${\vec{F}}_{k}^{l},{\vec{F}}_{k}^{s},\psi_{h},\psi_{l},\psi_{s},\lambda^{l},\lambda^{s},\left(\Delta_{1}^{h},\ldots \Delta_{\hat{h}}^{h}\right),\left(\Delta_{1}^{s},\ldots \Delta_{n}^{s}\right),\left(\Delta_{1}^{l},\ldots \Delta_{m}^{l}\right)$      **Output: ${\vec{c}}_{k}^{s},{\hat{\vec{c}}}_{k}^{s},{\vec{t}}_{k}^{s}$**1:${\vec{c}}_{k}^{l},{\hat{\vec{c}}}_{k}^{l}=findLocationClusters\left({\vec{F}}_{k}^{l},\lambda^{l},\left(\Delta_{1}^{h},\ldots \Delta_{\hat{h}}^{h}\right),\left(\Delta_{1}^{l},\ldots \Delta_{m}^{l}\right)\right)$ //Algorithm 12:${\vec{c}}_{k}^{s}=mapClusters\left({\vec{c}}_{k}^{l},{\hat{\vec{c}}}_{k}^{l},\psi_{l},\psi_{s}\right)$ //Algorithm 23:${\vec{c}}_{k}^{s},{\hat{\vec{c}}}_{k}^{s}=findSensorClusters\left({\vec{F}}_{k}^{s},{\vec{c}}_{k}^{s},\lambda^{s},\left(\Delta_{1}^{h},\ldots \Delta_{\hat{h}}^{h}\right),\left(\Delta_{1}^{s},\ldots \Delta_{n}^{s}\right)\right)$ //Algorithm 34:${\vec{t}}_{k}^{s}=assignTimeRef(|{\vec{F}}_{k}^{s}\left|,\psi_{s},\psi_{h}\right)$ //Algorithm 4

### 6.2. Inter-Day Pattern Discovery

The sensor feature vectors ${\vec{F}}_{k}^{s}$ for a day *k* are grouped into clusters ${\hat{\vec{c}}}_{k}^{s}$, where each cluster ${\hat{o}}_{w}^{s}$ represents a specific pattern ${\vec{p}}_{w}^{s}$. The identified patterns are frequent and repeatable between all days. In order to retain only interesting patterns, and group repeatable and frequent variations of patterns across all days, we use the Earth mover’s distance (EMD) [61] to evaluate the similarity between the patterns across all days. The EMD is also known as the Wasserstein distance, where it is a family of metrics used to compare the distributions based on the optimal transport problem.

**Figure 15:**
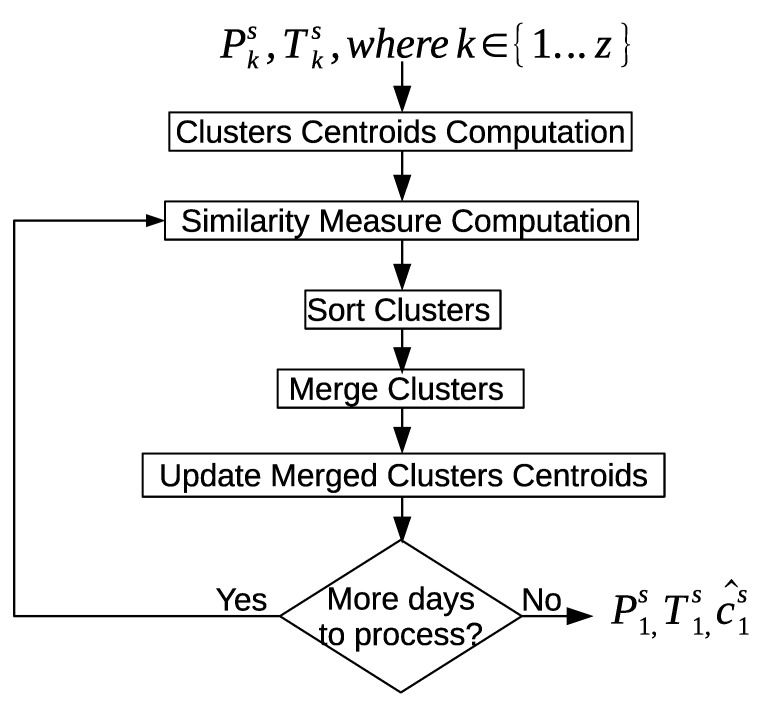
An overview of the inter-day discovery patterns algorithm.

Figure 15 shows an overview of the inter-day pattern discovery approach. First, the clusters centroids are computed for the sensor feature vectors and the time references of each day, and *z* is the number of days in the data set. Next, we use the EMD to measure the similarity between the clusters of the different days. According to the similarity scores of the clusters, the clusters are sorted. Furthermore, the clusters are merged according to the sorted similarity scores. The centroids of the merged clusters are updated. We keep processing more clusters from the different days till all the clusters are merged, and no further clusters merge. The clusters of all days will be merged into the first day. Therefore, ${\hat{\vec{c}}}_{1}^{s}$ will contain all the merged clusters between days, ${\vec{P}}_{1}^{s}$ will contain all the sensor feature vectors of all the merged clusters between days, and ${\vec{T}}_{1}^{s}$ will contain all the sensor time references of all the merged clusters between days.

#### 6.2.1. Cluster Centroids’ Computation

We compute the cluster centroid for ${\hat{o}}_{w}^{s}$ using its data ${\vec{p}}_{w}^{s}$. A centroid is a vector that contains one number for each variable, where each number is the mean of a variable for the observations in that cluster. We define ${\hat{\vec{p}}}_{w}^{s}$ to be the cluster centroid for a cluster ${\hat{o}}_{w}^{s}$. In our approach, we compute the mean duration for *N* sensors, and for the last two feature values we compute the mode. The mode of a sensor feature is the value that occurs most frequently in the collection. As explained in Section 5.2, the last two feature values represent whether a person left or entered the apartment. They are given constant values of $2\psi_{s}$. Otherwise, they are given values of zeroes. We do not want to manipulate their values by computing the mean; we want to keep their values constant across our calculations. Therefore, the mode operation is used:

(11) $${\hat{\vec{p}}}_{w}^{s}=(\frac{\sum_{c=1}^{a_{w}}d_{c}^{s_{1}}}{a_{w}},\ldots \frac{\sum_{c=1}^{a_{w}}d_{c}^{s_{N}}}{a_{w}},\;\mathrm{mode}(d_{1}^{S_{N+1}},\ldots d_{a_{w}}^{S_{N+1}}),\;\mathrm{mode}(d_{1}^{S_{N+2}},\ldots d_{a_{w}}^{S_{N+2}}))$$

We define ${\hat{\vec{P}}}_{k}^{s}=\left({\hat{\vec{p}}}_{1}^{s},{\hat{\vec{p}}}_{2}^{s}\ldots {\hat{\vec{p}}}_{\hat{n}}^{s}\right)$ to be the cluster centroid data for ${\hat{\vec{c}}}_{k}^{s}$ in a day *k*. Let ${\hat{t}}_{w}^{s}=\mathrm{mode}\left({\vec{u}}_{w}^{s}\right)$ be the most frequent time reference for a cluster ${\hat{o}}_{w}^{s}$ in ${\vec{u}}_{w}^{s}$. We define ${\hat{\vec{T}}}_{k}^{s}=\left({\hat{t}}_{1}^{s},{\hat{t}}_{2}^{s},\ldots {\hat{t}}_{\hat{n}}^{s}\right)$ to be the time reference centroid points for ${\hat{\vec{c}}}_{k}^{s}$ in a day *k*.

Next, we use ${\hat{\vec{P}}}_{k}^{s}$ and ${\hat{\vec{T}}}_{k}^{s}$ to compute the similarity between the clusters from the different days using EMD.

#### 6.2.2. Similarity Measure Computation

The EMD assesses the variation between two probability distribution domains in some dimensional attribute space, where a distance measure between individual attributes is referred to as the ground measure. The EMD moves the ground measure from single attributes to full probability distribution domains. Instinctively, given two probability distribution domains, the first probability distribution can be seen as a sand pile appropriately spread in a domain; the second probability distribution can be seen as a group of ground holes in the same domain of the first probability distribution. Then, the EMD evaluates the minimum number of moves required to fill the holes with sand. A unit of effort is defined here as moving a unit of sand by a unit of ground measure. As a special case, ${\hat{\vec{p}}}_{w}^{s}$ is a one-dimensional vector of values, the EMD can be efficiently computed by scanning the vector and keeping track of how much dirt needs to be transported between consecutive values.

**Figure 16:**
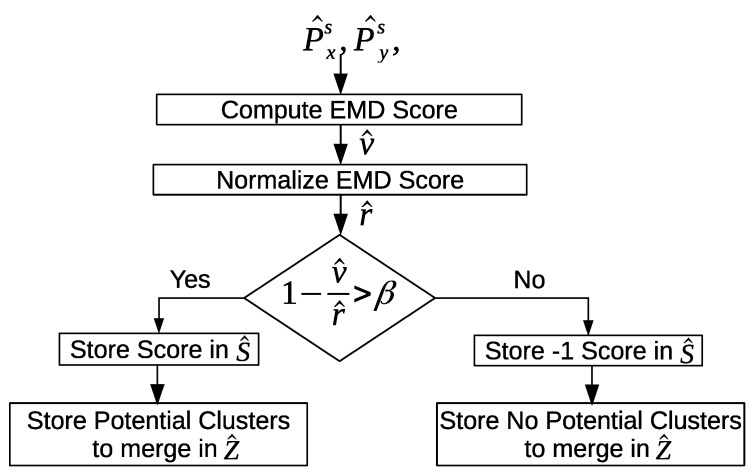
An overview of computing the similarity score using Earth mover’s distance (EMD).

Figure 16 shows an overview of measuring the similarity between the clusters from the different days using EMD. First, we compute the EMD value between ${\hat{\vec{p}}}_{x}^{s}$ from a day *i*, and ${\hat{\vec{p}}}_{y}^{s}$ from a day *j*. Then the EMD value is normalized using a normalization constant. The normalized EMD value is evaluated against a threshold; if the normalized EMD value is greater than the threshold, then the EMD score is stored, and clusters *x* and *y* are marked as potential clusters to merge. Otherwise, a constant value score is assigned and stored, and the two clusters are not considered as potential clusters to merge.

The similarity between two clusters is computed using EMD, as shown in Algorithm 6. Let $\hat{v}$ be the similarity value between cluster ${\hat{\vec{p}}}_{x}^{s}$ and cluster ${\hat{\vec{p}}}_{y}^{s}$. We define $\hat{r}$ to be the normalization constant, and the computation of $\hat{r}$ is shown in Algorithm 7. Then, $\hat{v}$ is normalized using $\hat{r}$ as follows:

(12) $$emd=1-\frac{\hat{v}}{\hat{r}}$$

**Algorithm 6** computeEMD.   **Input:**
${\hat{\vec{p}}}_{x}^{s},{\hat{\vec{p}}}_{y}^{s}$      **Output: $\hat{v}$**1:$\hat{v}=0,total\_sum=0,tmp\_sum=0$2:**for**c←1 to N+2 **do**3:  $tmp\_sum=d_{x}^{s_{c}}+tmp\_sum-d_{y}^{s_{c}}$
4:  total_sum=total_sum+tmp_sum
5:**end for**6:$\hat{v}=\left|total\_sum\right|$
**Algorithm 7** computeNormConstant.   **Input:**
$T_{s},N+2$      **Output: $\hat{r}$**1:$\hat{r}=0$2:**for**c←1 to N+2 **do**3:  ${\hat{a}}_{c}=0,{\hat{b}}_{c}=0$
4:**end for**5:${\hat{a}}_{1}=T_{s}$6:${\hat{b}}_{N+2}=2T_{s}$7:computeEMD($\hat{\vec{a}},\hat{\vec{b}},\hat{r})$ //Algorithm 6

Based on the emd value, the two clusters are marked as potential clusters to merge, if the score is greater than a threshold β, and the score is stored for later use in our approach. Otherwise, the two clusters are not considered as potential clusters to merge. We define $\hat{\vec{S}}$ to be the similarity matrix that will hold the normalized EMD similarity scores between the clusters of days *i* and *j*. We define $\hat{\vec{Z}}$ to be the matrix that will hold the cluster IDs for the potential clusters to merge. The similarity matrix $\hat{\vec{S}}$ is initialized with values of –1, and that means no potential clusters are considered for merging. Similarly, $\hat{\vec{Z}}$ is initialized with values of –1 in order to indicate no potential clusters to merge. The numbers of rows and columns for $\hat{\vec{S}}$ and $\hat{\vec{Z}}$ depend on the numbers of clusters in days *i* and *j*. The similarity measure computation between days *i* and *j* is shown in Algorithm 8. We define $|{\hat{\vec{P}}}_{i}^{s}|$ to be the number of clusters for a day *i*. Similarly, we define $|{\hat{\vec{P}}}_{j}^{s}|$ to be the number of clusters for a day *j*. Next, we will sort the potential clusters to merge in $\hat{\vec{Z}}$ using $\hat{\vec{S}}$.
**Algorithm 8** computeSimilarity.   **Input:**
${\hat{\vec{P}}}_{i}^{s},{\hat{\vec{c}}}_{j}^{s},{\hat{\vec{P}}}_{j}^{s},{\hat{\vec{T}}}_{i}^{s},{\hat{\vec{T}}}_{j}^{s},T_{s},N+2$      **Output: $\hat{\vec{S}},\hat{\vec{Z}}$**1:$\hat{r}=computeNormConstant\left(T_{s},N+2\right)$ //Algorithm 72:**for**h←1 to $|{\hat{\vec{P}}}_{i}^{s}|$ **do**3:  **for** g←1 to $|{\hat{\vec{P}}}_{j}^{s}|$ **do**4:    ${\hat{S}}_{\left(h,g\right)}=-1$
5:    ${\hat{Z}}_{\left(h,g\right)}=-1$6:  **end for**7:**end for**8:**for**h←1 to $|{\hat{\vec{P}}}_{i}^{s}|$ **do**9:  **for** g←1 to $|{\hat{\vec{P}}}_{j}^{s}|$ **do**10:    **if** ${\hat{t}}_{h}^{s}\neq {\hat{t}}_{g}^{s}$ **then**11:      break12:    **end if**13:    $\hat{v}=computeEMD\left({\hat{\vec{p}}}_{h}^{s},{\hat{\vec{p}}}_{g}^{s}\right)$ //Algorithm 614:    $emd=1-\frac{\hat{v}}{\hat{r}}$15:    **if** emd>β **then**
16:      ${\hat{S}}_{\left(h,g\right)}=emd$
17:      ${\hat{Z}}_{\left(h,g\right)}={\hat{o}}_{g}^{s}$
18:    **end if**19:  **end for**20:**end for**

#### 6.2.3. Cluster Sorting

**Figure 17:**
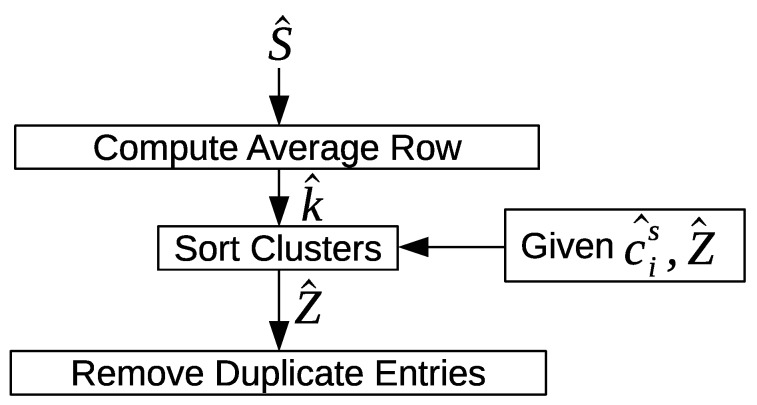
An overview of the cluster sorting approach.

Each row in $\hat{\vec{S}}$ and $\hat{\vec{Z}}$ represents a cluster in a day *i*, and each column represents a cluster in a day *j*. Therefore, a single row represents all the potential clusters that can be merged in a day *j* with a given cluster in a day *i*. Figure 17 shows an overview of the cluster sorting approach. First, the average emd similarity score for each row is computed in $\hat{\vec{S}}$. Next, the rows in $\hat{\vec{Z}}$ are sorted in a descending order according to the average emd similarity score per row. A column value from $\hat{\vec{Z}}$ should only belong to the row with the best average emd similarity score in $\hat{\vec{Z}}$. This is the current state of $\hat{\vec{Z}}$, because a column value could belong to multiple rows. In this case, the duplicate entries should be removed, and only the row with the best average emd similarity score should keep the column value.

We define $\hat{\vec{k}}=\left({\hat{k}}_{1},{\hat{k}}_{2},\ldots {\hat{k}}_{|{\hat{\vec{P}}}_{i}^{s}|}\right)$ to be the average emd similarity scores per row for $\hat{\vec{S}}$. Algorithm 9 shows how $\hat{\vec{k}}$ is computed. The row entries in $\hat{\vec{Z}}$ are sorted in a descending order according to the average emd similarity scores in $\hat{\vec{k}}$, as shown in Algorithm 10. Finally, the duplicate entries are removed from all rows in $\hat{\vec{Z}}$, and only one instance is kept for the rows with the best average emd similarity score. Let $\hat{\vec{l}}=\left({\hat{l}}_{1},{\hat{l}}_{2},\ldots {\hat{l}}_{|{\hat{\vec{P}}}_{i}^{s}\left|\times \right|{\hat{\vec{P}}}_{j}^{s}|}\right)$ be a temporary vector holder for the column values for a given row in $\hat{\vec{Z}}$. Each row in $\hat{\vec{Z}}$ is scanned, and the duplicate entries are marked with values of –1 if the column values already belong to a row with the best average emd similarity score. Otherwise, the column values are considered unique, and they should belong to the row that is being scanned. This is illustrated in Algorithm 11.

Next, the clusters between days *i* and *j* are merged according to $\hat{\vec{Z}}$.
**Algorithm 9** computeAverageRow.   **Input:**
$\hat{\vec{S}}$      **Output: $\hat{\vec{k}}$**1:**for**c←1 to $|{\hat{\vec{P}}}_{i}^{s}|$ **do**2:  ${\hat{k}}_{c}=0$3:**end for**4:**for**h←1 to $|{\hat{\vec{P}}}_{i}^{s}|$ **do**5:  avg=0,size=06:  **for** g←1 to $|{\hat{\vec{P}}}_{j}^{s}|$ **do**7:    **if** ${\hat{S}}_{\left(h,g\right)}\neq -1$ **then**8:      $avg=avg+{\hat{S}}_{\left(h,g\right)}$
9:      size=size+1
10:    **end if**10:  **end for**12:  ${\hat{k}}_{h}=\frac{avg}{size}$
13:**end for**
**Algorithm 10** sortClusters.   **Input:**
$\hat{\vec{Z}},{\hat{\vec{c}}}_{i}^{s},\hat{\vec{k}}$      **Output: $\hat{\vec{Z}},{\hat{\vec{c}}}_{i}^{s}$**1:$score=0,index=0,\vec{row},flag=1$2:**for**h←1 to $|{\hat{\vec{P}}}_{i}^{s}|$ **do**3:  flag=0
4:  **for** $g\leftarrow 2;\;g<|{\hat{\vec{P}}}_{i}^{s}|\wedge flag;\;g\leftarrow g+1$ **do**5:    **if** ${\hat{k}}_{g}<{\hat{k}}_{g-1}$ **then**6:      $index={\hat{o}}_{g-1}^{s}$ //swap elements7:      ${\hat{o}}_{g-1}^{s}={\hat{o}}_{g}^{s}$
8:      ${\hat{o}}_{g}^{s}=index$
9:      $\vec{row}={\hat{\vec{Z}}}_{\left(g-1,*\right)}$ //swap elements10:      ${\hat{\vec{Z}}}_{\left(g-1,*\right)}={\hat{\vec{Z}}}_{\left(g,*\right)}$
11:      ${\hat{\vec{Z}}}_{\left(g,*\right)}=\vec{row}$
12:      flag=1 // indicates that a swap occurred.13:    **end if**14:  **end for**15:**end for**
**Algorithm 11** removeDuplicateEntries.   **Input:**
$\hat{\vec{Z}}$      **Output: $\hat{\vec{Z}}$**1:**for**g←1 to $|{\hat{\vec{P}}}_{i}^{s}\left|\times \right|{\hat{\vec{P}}}_{j}^{s}|$ **do**2:  ${\hat{l}}_{g}=-1$3:**end for**4:**for**h←1 to $|{\hat{\vec{P}}}_{i}^{s}|$ **do**5:  **for** g←1 to $|{\hat{\vec{P}}}_{j}^{s}|$ **do**6:    flag=07:    **For** c←1 to $|{\hat{\vec{P}}}_{i}^{s}\left|\times \right|{\hat{\vec{P}}}_{j}^{s}|$ **do**8:      **if** ${\hat{l}}_{c}={\hat{Z}}_{\left(h,g\right)}\wedge {\hat{l}}_{c}\neq -1$ **then** //column value is not unique9:        ${\hat{Z}}_{\left(h,g\right)}=-1$
10:        flag=111:      **end if**12:    **end for**13:    **if** flag=0 **then** // column value is unique14:      ${\hat{l}}_{g}={\hat{Z}}_{\left(h,g\right)}$
15:    **end if**16:  **end for**17:**end for**

#### 6.2.4. Cluster Merging

The information of the potential clusters to be merged between days *i* and *j* is encoded in $\hat{\vec{Z}}$. First, we merge the potential cluster data, such as the sensor feature vectors and the sensor time references from day *j* with day(s) *i*. There could be some clusters that are not considered as potential clusters to merge from day *j* to day(s) *i*. In this case, the cluster data from day *j* is appended to day(s) *i*. We define $\hat{b}$ to be the number of clusters in a day *j* that are not considered to be merged with any of the clusters in day(s) *i*. The size of the sensor feature vectors of day(s) *i* is expanded as follows. ${\vec{P}}_{i}^{s}=\left({\vec{p}}_{1}^{s},{\vec{p}}_{2}^{s},\ldots {\vec{p}}_{\hat{n}+\hat{b}}^{s}\right)$, where $\hat{n}$ is the number of clusters. Similarly, the size of the sensor time references of day(s) *i* is expanded as follows. ${\vec{T}}_{k}^{s}=\left({\vec{u}}_{1}^{s},{\vec{u}}_{2}^{s},\ldots {\vec{u}}_{\hat{n}+\hat{b}}^{s}\right)$. The clusters’ IDs are expanded as follows: ${\hat{\vec{c}}}_{k}^{s}=\left({\hat{o}}_{1}^{s},{\hat{o}}_{2}^{s},\ldots {\hat{o}}_{\hat{n}+\hat{b}}^{s}\right)$. We use ⌢ notation to refer to the concatenation of two sequences. Algorithm 12 illustrates the cluster merging approach.
**Algorithm 12** mergeClusters.   **Input:**
$\hat{\vec{Z}},{\vec{P}}_{i}^{s},{\vec{T}}_{i}^{s},{\hat{\vec{c}}}_{i}^{s},{\vec{P}}_{j}^{s},{\vec{T}}_{j}^{s},{\hat{\vec{c}}}_{j}^{s}$      **Output: ${\vec{P}}_{i}^{s},{\vec{T}}_{i}^{s},{\hat{\vec{c}}}_{i}^{s}$**1:**for**h←1 to $|{\hat{\vec{P}}}_{j}^{s}|$ **do**2:  **for** g←1 to $|{\hat{\vec{P}}}_{j}^{s}|$ **do**3:    **if** ${\hat{Z}}_{\left(h,g\right)}={\hat{o}}_{g}^{s}\wedge {\hat{Z}}_{\left(h,g\right)}\neq -1$ **then** //merge cluster data from day j to day i4:      ${\vec{p}}_{h}^{s}={\vec{p}}_{h}^{s}⌢{\vec{p}}_{g}^{s}$
5:      ${\vec{u}}_{h}^{s}={\vec{u}}_{h}^{s}⌢{\vec{u}}_{g}^{s}$
6:      ${\hat{o}}_{g}^{s}=-1$ //mark cluster in a day j as merged7:    **end if**8:  **end for**9:**end for**10:$h=|{\hat{\vec{P}}}_{i}^{s}|+1$11:**for**g←1 to $|{\hat{\vec{P}}}_{j}^{s}|$ **do**12:  **if** ${\hat{o}}_{g}^{s}\neq -1$ **then** // cluster not merged13:    ${\hat{o}}_{h}^{s}={\hat{o}}_{g}^{s}$ // add cluster data from day j to day i14:    ${\vec{p}}_{h}^{s}={\vec{p}}_{g}^{s}$
15:    ${\vec{u}}_{h}^{s}={\vec{u}}_{g}^{s}$
16:    h=h+1
17:  **end if**18:**end for**

Algorithm 13 summarizes the algorithms of the inter-day pattern discovery approach. The clusters of all days are merged into the first day. Therefore, ${\hat{\vec{c}}}_{1}^{s}$ will contain all the merged clusters across all days, ${\vec{P}}_{1}^{s}$ will contain all the sensor feature vectors of all the merged clusters across all days, and ${\vec{T}}_{1}^{s}$ will contain all the sensor time references of all the merged clusters across all days. Let $\hat{N}$ be the size of ${\hat{\vec{c}}}_{1}^{s}$, ${\vec{P}}_{1}^{s}$, and ${\vec{T}}_{1}^{s}$ after merging the clusters across all days.
**Algorithm 13** computeInterClusters.   **Input:**
$\left({\vec{P}}_{1}^{s},\ldots {\vec{P}}_{z}^{s}\right),\left({\vec{T}}_{1}^{s},\ldots {\vec{T}}_{z}^{s}\right),\left({\hat{\vec{c}}}_{1}^{s},\ldots {\hat{\vec{c}}}_{z}^{s}\right)$      **Output: ${\vec{P}}_{1}^{s},{\vec{T}}_{1}^{s},{\hat{\vec{c}}}_{1}^{s}$**1:**for**a←1 to *z* **do**2:  compute cluster centroids for ${\vec{P}}_{a}^{s}$ and ${\vec{T}}_{a}^{s}$ as in Equation (11)3:**end for**4:**for**a←2 to *z* **do**5:  $\hat{\vec{S}},\hat{\vec{Z}}=computeSimilarity\left({\vec{P}}_{1}^{s},{\vec{T}}_{1}^{s},{\hat{\vec{c}}}_{1}^{s},{\vec{P}}_{a}^{s},{\vec{T}}_{a}^{s},{\hat{\vec{c}}}_{a}^{s},T_{s},N+2\right)$ // Algorithm 86:  $\hat{\vec{k}}=computeAverageRow(\hat{\vec{S}})$ //Algorithm 97:  $\hat{\vec{Z}},{\hat{\vec{c}}}_{1}^{s}=sortClusters\left(\hat{\vec{Z}},{\hat{\vec{c}}}_{1}^{s},\hat{\vec{k}}\right)$ //Algorithm 108:  $\hat{\vec{Z}}=removeDuplicateEntries\left(\hat{\vec{Z}}\right)$ //Algorithm 119:  ${\vec{P}}_{1}^{s},{\vec{T}}_{1}^{s},{\hat{\vec{c}}}_{1}^{s}=mergeClusters\left(\hat{\vec{Z}},{\vec{P}}_{1}^{s},{\vec{T}}_{1}^{s},{\hat{\vec{c}}}_{1}^{s},{\vec{P}}_{a}^{s},{\vec{T}}_{a}^{s},{\hat{\vec{c}}}_{a}^{s}\right)$ //Algorithm 1210:  compute merged cluster centroids for ${\vec{P}}_{1}^{s}$ and ${\vec{T}}_{1}^{s}$ as in Equation (11)11:**end for**

### 6.3. Cluster Refinement

After grouping the general frequent clusters across all days, we refine the clusters in order to get a more compressed representation. Though we grouped the redundant cluster variations in order to retain interesting ones in the previous step, their categorization is still merely based on sensors’ time durations and time references. Two similar clusters with a small degree of trajectory differences may trigger various kinds of sensors, and they can be treated as different clusters even if the clusters show high similarity in time references, and sensors’ time durations. In order to solve this problem, we introduce a cluster refinement algorithm as an extra step to our framework. The cluster refinement algorithm groups the clusters using three properties: (1) sensors’ structure, (2) time references, and (3) sensors’ time duration. The cluster refinement also tackles the situation when too many clusters have been discovered, where a large number of clusters makes it challenging to examine the true number of important clusters.

Our cluster refinement approach is equivalent to the standard hierarchical clustering methods [62], although it does not build a total hierarchy. There are two ways to build up the hierarchy in this clustering algorithm. The “bottom-up” way: each pattern is considered in its own cluster, and two patterns are merged into one cluster and then gradually keep merging the rest of the patterns until all patterns are merged into one cluster. This technique is referred to “agglomerative”. In the “top-down” way: all patterns are considered in one cluster, and a cluster division is done through a repeated approach until all patterns are divided into a certain predefined number of clusters. This technique is referred to “divisive”. Our cluster refinement approach is of agglomerative type. In our approach, we do not proceed until all the patterns are merged into one cluster; rather, the cluster refinement proceeds until the similarity value between the two closest clusters falls under a threshold τ. This provides us with a group of clusters at the top level of the hierarchy. Using such a clustering refinement method, the individual is no longer required to choose the number of clusters beforehand.

**Figure 18:**
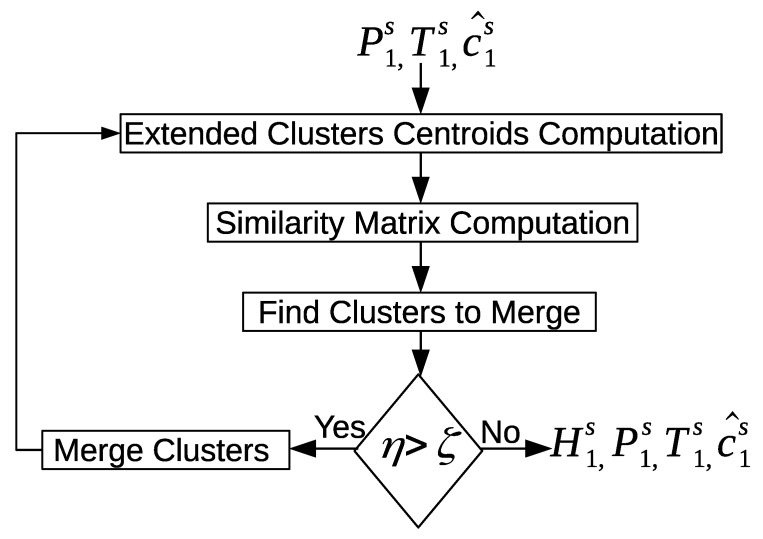
An overview of the cluster refinement approach.

Figure 18 shows an overview of the cluster refinement approach. First, the clusters centroids are computed. Next, the similarity matrix of the clusters is computed using a similarity measure. The maximum similarity value is extracted from the similarity matrix along the two potential clusters to merge. The maximum similarity value between the two potential clusters is evaluated against a threshold; if the value is larger than the threshold, then the two potential clusters are merged. Otherwise, the algorithm stops merging further clusters.

#### 6.3.1. Extended Cluster Centroid Computation

In the inter-day discovery patterns approach, we used only the sensors’ time duration to compute the clusters’ centroids. In the cluster refinement algorithm, we use three properties; namely, the sensors’ time duration, the time references, and the sensors’ structure to compute the clusters’ centroids for the refinement. The sensors’ structure is defined as the key triggered sensors during the user’s activity or movement within a time interval $\Delta_{i}^{s}$.

We define ${\hat{\vec{P}}}_{1}^{s}=\left({\hat{\vec{p}}}_{1}^{s},{\hat{\vec{p}}}_{2}^{s},\ldots {\hat{\vec{p}}}_{\hat{N}}^{s}\right)$ to be the first property in the cluster centroids data for clusters ${\hat{\vec{c}}}_{1}^{c}$. We refer to ${\hat{\vec{P}}}_{1}^{s}$ as the sensors’ time duration property, and it is computed as explained in Section 6.2.1. We define ${\hat{\vec{T}}}_{1}^{s}=\left({\hat{t}}_{1}^{s},{\hat{t}}_{2}^{s},\ldots {\hat{t}}_{\hat{N}}^{s}\right)$ to be the second property in the cluster centroids’ data for clusters ${\hat{\vec{c}}}_{1}^{s}$. We refer to ${\hat{\vec{T}}}_{1}^{s}$ as the time references property; it is computed as explained in Section 6.2.1.

Using ${\hat{\vec{P}}}_{1}^{s}$, we compute the sensors’ structure. Let ${\hat{\vec{y}}}_{i}^{s}=\left(\alpha_{i}^{1},\alpha_{i}^{2},\ldots \alpha_{i}^{{\hat{q}}_{i}}\right)$ be the sensors’ structure for a time interval $\Delta_{i}^{s}$, where ${\hat{q}}_{i}$ is the number of key triggered sensors’ in ${\hat{\vec{y}}}_{i}^{s}$, and $\alpha_{i}^{m}$ is an index of the sensor ID. Algorithm 14 shows how the sensor structure is computed. Each value in ${\hat{\vec{p}}}_{i}^{s}$ is checked; if the value is larger than zero, then the index of the sensor ID is added to ${\hat{\vec{y}}}_{i}^{s}$. Otherwise, no value is added. A non-zero value means that a sensor was activated for a time duration of $d_{i}^{s_{c}}$.

We define ${\vec{\hat{z}}}_{w}^{s}=\left({\hat{\vec{y}}}_{1}^{s},{\hat{\vec{y}}}_{2}^{s},\ldots {\hat{\vec{y}}}_{{\hat{j}}_{w}}^{s}\right)$ to be the group of sensors’ structure that belongs to a cluster ${\hat{o}}_{w}^{s}$, where ${\hat{j}}_{w}$ is the number of the sensors’ structure that belongs to ${\vec{\hat{z}}}_{w}^{s}$ and $w\in \{1,2,\ldots \hat{N}\}$. We define ${\vec{H}}_{1}^{s}=\left({\vec{\hat{z}}}_{1}^{s},{\vec{\hat{z}}}_{2}^{s},\ldots {\vec{\hat{z}}}_{\hat{N}}^{s}\right)$ to be the third property in the cluster centroids’ data for clusters ${\hat{\vec{c}}}_{1}^{s}$. We refer to ${\vec{H}}_{1}^{s}$ as the sensors’ structure property.
**Algorithm 14** computeSensorsStructure.   **Input:**
${\hat{\vec{p}}}_{i}^{s}$      **Output: ${\hat{\vec{y}}}_{i}^{s}$**1:index=02:**for**m←1 to *N* **do**3:  **if** $d_{i}^{s_{m}}>0$ **then**4:    $\alpha_{i}^{index}=m$
5:    index=index+1
6:  **end if**7:**end for**

#### 6.3.2. Similarity Matrix Computation

**Figure 19:**
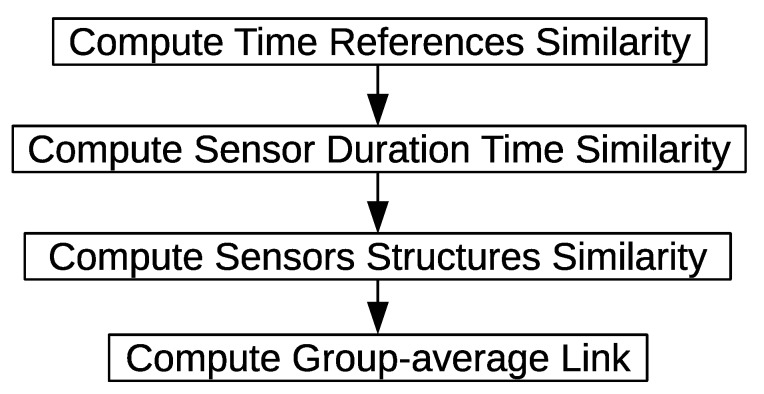
An overview of the similarity matrix computation.

A cluster centroid is represented by three properties: (1) the time reference ${\hat{t}}_{i}^{s}$; (2) the sensor time duration $d_{i}^{s_{\hat{u}}}$; and (3) the sensor structure ${\hat{\vec{y}}}_{i}^{s}$. We use a group-average link method [62] to compute the similarity matrix. Figure 19 shows an overview of computing the similarity matrix. First, the time references similarity between cluster ${\hat{o}}_{i}^{s}$ and cluster ${\hat{o}}_{j}^{s}$ is computed. The sensors’ time duration similarity between cluster ${\hat{o}}_{i}^{s}$ and cluster ${\hat{o}}_{j}^{s}$ is computed. The sensors’ structure similarity between cluster ${\hat{o}}_{i}^{s}$ and cluster ${\hat{o}}_{j}^{s}$ is computed. Finally, the average similarity values of the three properties is computed and assigned to the similarity matrix.

Let $\vec{Z}$ be the similarity matrix with a size of $\hat{N}\times \hat{N}$, where $\hat{N}$ is the number of clusters across all days. We define $\gamma^{s}\left(i,j\right)$ to be the time reference similarity value between cluster ${\hat{o}}_{i}^{s}$ and cluster ${\hat{o}}_{j}^{s}$. The time reference ${\hat{t}}_{i}^{s}$ of cluster ${\hat{o}}_{i}^{s}$ is represented in an angular measure unit $\phi_{i}^{s}$ computed using the radian measure unit as an alternative to the linear representation. This enables the differences in time to appear accurately (1:00 AM will be nearer to 22:00 PM than 18:00 PM). The angular time of the time reference ${\hat{t}}_{i}^{s}$ for cluster ${\hat{o}}_{i}^{s}$ is computed as follows:

(13) $$\phi_{i}^{s}=\left(\frac{{\hat{t}}_{i}^{s}}{24}\times 360\right)\left(\frac{\pi }{180}\right)$$

Similarly, the time reference ${\hat{t}}_{j}^{s}$ of cluster ${\hat{o}}_{i}^{s}$ is represented in an angular form $\phi_{j}^{s}$. The time reference attribute can help us to differentiate between certain routines, such as late breakfast versus late lunch. The time reference similarity value is computed as follows:

(14) $$\gamma^{s}\left(i,j\right)=1-\frac{|\phi_{i}^{s}-\phi_{j}^{s}|}{2\pi },$$

We define $\tau^{s}\left(i,j\right)$ to be the sensor time duration similarity value between cluster ${\hat{o}}_{i}^{s}$ and cluster ${\hat{o}}_{j}^{s}$. It is computed as follows:

(15) $$\tau^{s}\left(i,j\right)=\max_{\begin{array}{c} d_{i}^{s_{c}}\in {\hat{\vec{f}}}_{i}^{s}\\ d_{j}^{s_{c}}\in {\hat{\vec{f}}}_{j}^{s} \end{array}}\left(\frac{|d_{i}^{s_{c}}-d_{j}^{s_{c}}|}{\max(d_{i}^{s_{c}},d_{j}^{s_{c}})}\right)$$

We define $\varrho^{s}\left(i,j\right)$ to be the sensor structure similarity value between cluster ${\hat{o}}_{i}^{s}$ and cluster ${\hat{o}}_{j}^{s}$. The sensor structure similarity is calculated as in Equation (16) using the Jaccard similarity measure [62]:

(16) $$\varrho^{s}\left(i,j\right)=\frac{{\hat{\vec{y}}}_{i}\cap {\hat{\vec{y}}}_{j}}{{\hat{\vec{y}}}_{i}\cup {\hat{\vec{y}}}_{j}}.$$

Finally, the average similarity value between cluster ${\hat{o}}_{i}^{s}$ and cluster ${\hat{o}}_{j}^{s}$ is computed as follows:

(17) $$Z_{\left(i,j\right)}=\frac{\gamma^{s}\left(i,j\right)+\tau^{s}\left(i,j\right)+\varrho^{s}\left(i,j\right)}{3}$$

where we normalize $Z_{\left(i,j\right)}$ by 3 in order to fall within the range [0,…,1]. Next, we find the two best matching clusters to merge.

#### 6.3.3. Finding Potential Clusters

We are interested in finding the two best clusters to merge according to the best similarity value between clusters in $\vec{Z}$. Our approach intends to find a global similarity value in $\vec{Z}$ between two clusters. Algorithm 15 shows how the two best matching clusters to merge are found.
**Algorithm 15** findPotentialClusters.   **Input:**
$\vec{Z}$      **Output: $\eta ,r^{*}$, $c^{*}$**1:${\hat{\vec{d}}}^{r}\leftarrow 0,{\hat{\vec{d}}}^{c}\leftarrow 0,{\hat{\vec{d}}}^{s}\leftarrow 0$2:$r^{*}=0,c^{*}=0,b^{*}=0,\eta =0$3:**for**i←1 to $\hat{N}$ **do**4:  ${\hat{d}}_{i}^{r}=i$
5:  ${\hat{d}}_{i}^{s}=\max\left(\vec{Z}(i,*\right))$
6:  ${\hat{d}}_{i}^{c}=i^{*}$
7:**end for**8:$\eta =\max({\hat{\vec{d}}}^{s})$9:$r^{*}={\hat{d}}_{b^{*}}^{r}$10:$c^{*}={\hat{d}}_{b^{*}}^{c}$

We define ${\hat{\vec{d}}}^{r}=\left({\hat{d}}_{1}^{r},{\hat{d}}_{2}^{r},\ldots {\hat{d}}_{\hat{N}}^{r}\right)$ to hold the rows positions in $\vec{Z}$. We define ${\hat{\vec{d}}}^{c}=\left({\hat{d}}_{1}^{c},{\hat{d}}_{2}^{c},\ldots {\hat{d}}_{\hat{N}}^{c}\right)$ to hold the positions of the maximum similarity values of the rows in $\vec{Z}$. We define ${\hat{\vec{d}}}^{s}=\left({\hat{d}}_{1}^{s},{\hat{d}}_{2}^{s},\ldots {\hat{d}}_{\hat{N}}^{s}\right)$ to hold the maximum similarity values of the rows in $\vec{Z}$. Next, each row in $\vec{Z}$ is scanned to extract the maximum similarity value, and the position of the maximum similarity value as well. Let $i^{*}$ be the position of the maximum similarity value for a given row *i* in $\vec{Z}$. The row position, the maximum similarity value position, and the maximum similarity value are kept in ${\hat{\vec{d}}}^{r}$, ${\hat{\vec{d}}}^{c}$, and ${\hat{\vec{d}}}^{s}$ respectively.

Let η be a global similarity value, and it can be found by scanning ${\hat{\vec{d}}}^{s}$. Let $b^{*}$ be the corresponding position of the global similarity value of η. Then $b^{*}$ can be used to extract the positions of the two best matching clusters to merge from ${\hat{\vec{d}}}^{r}$, and ${\hat{\vec{d}}}^{c}$. We define $r^{*}$ and $c^{*}$ to be the positions of the two best matching clusters to merge. Next, we will merge the two best matching clusters according to the global similarity value η.

#### 6.3.4. Potential Cluster Merging

After having found the global similarity value, and the two best matching positions of the clusters to merge, the global similarity value η is evaluated against the cluster refinement threshold ζ. If η is larger than ζ, then the two best matching clusters $o_{r^{*}}^{s}$ and $o_{c^{*}}^{s}$ are merged. Otherwise, no further cluster refinement. Algorithm 16 shows how the two best matching clusters are merged. First, the sensor feature vectors ${\vec{p}}_{r^{*}}^{s}$ of cluster $o_{r^{*}}^{s}$ are merged with the sensor feature vectors ${\vec{p}}_{c^{*}}^{s}$ of cluster $o_{c^{*}}^{s}$. Next, the time references ${\vec{u}}_{r^{*}}^{s}$ of cluster $o_{r^{*}}^{s}$ are merged with the time references ${\vec{u}}_{c^{*}}^{s}$ of cluster $o_{c^{*}}^{s}$. The sensors’ structure ${\vec{z}}_{r^{*}}^{s}$ of cluster $o_{r^{*}}^{s}$ is merged with the sensors’ structure ${\vec{z}}_{c^{*}}^{s}$ of cluster $o_{c^{*}}^{s}$. Finally, $o_{c^{*}}^{s}$ is removed from ${\hat{\vec{c}}}_{1}^{s}$, and the number of clusters is decreased by $\hat{N}=\hat{N}-1$.
**Algorithm 16** mergePotentialClusters.   **Input:**
$\eta ,\zeta ,r^{*},c^{*},{\vec{P}}_{1}^{s},{\vec{T}}_{1}^{s},{\vec{H}}_{1}^{s},{\hat{\vec{c}}}_{1}^{s}$      **Output: ${\vec{P}}_{1}^{s},{\vec{T}}_{1}^{s},{\vec{H}}_{1}^{s},{\hat{\vec{c}}}_{1}^{s}$**1:**if**η>ζ**then**2:  ${\vec{p}}_{r^{*}}^{s}={\vec{p}}_{r^{*}}^{s}⌢{\vec{p}}_{c^{*}}^{s}$
3:  ${\vec{u}}_{r^{*}}^{s}={\vec{u}}_{r^{*}}^{s}⌢{\vec{u}}_{c^{*}}^{s}$
4:  ${\vec{z}}_{r^{*}}^{s}={\vec{z}}_{r^{*}}^{s}⌢{\vec{z}}_{c^{*}}^{s}$
5:  remove ${\hat{o}}_{c^{*}}^{s}$ from ${\hat{\vec{c}}}_{1}^{s}$6:  update $\hat{N}=\hat{N}-1$7:**end if**

We get a more compact and a more compressed representation of each cluster, after merging all the potential clusters using the cluster refinement approach. We will refer to each cluster in ${\hat{\vec{c}}}_{1}^{s}$ as a pattern by itself. The sensors’ time duration ${\vec{P}}_{1}^{s}$, the time references ${\vec{T}}_{1}^{s}$, and the sensors’ structure ${\vec{H}}_{1}^{s}$ represent the cluster data of the patterns (clusters) ${\hat{\vec{c}}}_{1}^{s}$.

## 7. Activity Recognition

We want to use the set of clusters to recognize the future executions of the discovered pattern. This will allow the smart environment to track each discovered pattern and determine if an individual’s routine is being maintained. In the high-level processing step, our approaches identified the key patterns data that likely belong together and appear with enough frequency and regularity to comprise a discovered pattern that can be tracked and analyzed. We want to assign descriptive, human-readable labels for the clusters. In order to do that, we use the externally provided annotations of the data sets to assign meaningful class labels to each cluster. We define $\xi_{i}^{s}$ to be the label that is being assigned to one of the points of the cluster data, where a cluster data point is the sensor feature vector ${\vec{f}}_{i}^{s}$, the time reference ${\hat{t}}_{i}^{s}$, and the sensor structure ${\hat{\vec{y}}}_{i}^{s}$. Let $\Xi_{m}^{s}=\left(\xi_{1}^{s},\xi_{2}^{s},\ldots \xi_{{\hat{g}}_{m}}^{s}\right)$ be the group of labels that are being assigned to a cluster ${\hat{o}}_{m}^{s}$, where ${\hat{g}}_{m}$ is the number of labels in ${\hat{\xi }}_{m}^{s}$, and the cluster data points are the sensor feature vectors ${\vec{p}}_{m}^{s}$, the time references ${\vec{u}}_{m}^{s}$, and the sensors’ structure ${\hat{\vec{z}}}_{m}^{s}$. A semantic label ${\hat{\xi }}_{m}^{s}$ is given to a cluster ${\hat{o}}_{m}^{s}$ by selecting the label that appears most frequently in $\Xi_{m}^{s}$ as follows:

(18) $${\hat{\xi }}_{m}^{s}=\mathrm{mode}\left(\Xi_{m}^{s}\right)$$

**Figure 20:**
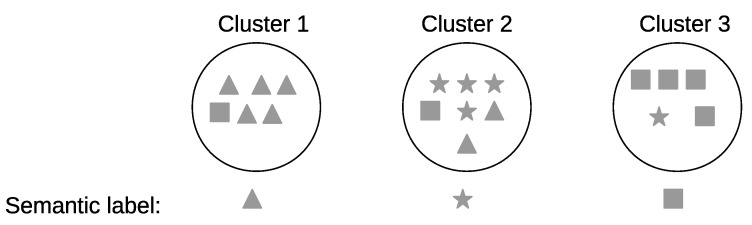
Illustration example to assign class labels to clusters. The most occurring class label inside a cluster is chosen as the semantic label for that cluster.

We define ${\hat{\Xi }}_{1}^{s}=\left({\hat{\xi }}_{1}^{s},{\hat{\xi }}_{2}^{s},\ldots {\hat{\xi }}_{\hat{N}}^{s}\right)$ to be the most frequent labels that are being assigned to clusters ${\hat{\vec{c}}}_{1}^{s}$, where the cluster data points are the sensor vector features ${\vec{P}}_{1}^{s}$, the time references ${\vec{T}}_{1}^{s}$, and the sensors’ structure ${\vec{H}}_{1}^{s}$. A cluster is considered as a good representation of its labels and data when all the labels are from the same class. Figure 20 shows an illustration of examples of semantic labels that are being assigned to clusters. The resulting set of cluster centroids represents the activities that we will model, recognize, and track.

## 8. Experiments

### 8.1. Data Set

We evaluated the performances of our methods on two public data sets captured in real-life settings from two apartments during seven-month and three-month periods. The data sets were collected in the CASAS smart home, a project of Washington State University, with full-time residents [32,60]. The two data sets are code-named “Milan” and “Aruba”. In case of “Milan”, 31 motion sensors and one door sensor were deployed at various locations and 15 activities were performed for 81 days. The residents in the home were a woman and a dog. For “Aruba”, 31 motion sensors and three door sensors were deployed and 12 activities were performed for 220 days. The resident in the home was a woman. The floor plan and sensor layout for the two apartments are shown in Figure 4. These data are all represented as a sequence of time-stamped sensor data, as shown in Figure 3. A detailed description of the data sets and annotation method can be found in [32,60].

In order to be capable of evaluating the outcomes of our methods, each of the data sets was labeled with predefined routines of interest for the corresponding occupants and apartments. Those routines are representative activities used in the validation of smart home monitoring systems. Table 3 shows the characteristics of “Aruba” and “Milan” data sets.

Most of the existing activity learning methods exclude the sensor data that are not in agreement with any of the known routine classes. To elaborate more, training activity models on certain routines such as “sleep”, “relax”, and “work” could lead to evaluating data that are consistent only with these three routines. However this does not reflect the real-life setting of an environment, where data can be correlated with other clear routines such as “cook” or “eat”, or switching between different routines. Normally, data from “other activity” show a tendency to impact on the real-life setting data, as shown by the great number of sensor events relating to the “other activity” class in Table 3. Current activity learning methods regularly exclude the existence of these “other activity” classes while validating the effectiveness of the methods. In this paper, we include the sensor events from these “other activity” classes also in order to define the utmost naturalistic activity learning performance.

### 8.2. Setup

We used the whole data sets for the evaluation. We split the data sets using the “leave-one-day-out” strategy; therefore, the sensor readings of one day are used for testing and the remaining days are used to form the clusters with their corresponding semantic labels. For classifying the label of a test time interval of sensor events, we use two approaches: (1) classification by cluster; and (2) classification by model.

In the first approach, for a testing day, we use a sensor time interval length of 60 s to divide the entire sensor event sequence into an equal size of time intervals. This time interval length is long enough to be discriminative and short enough to provide high accuracy labeling results. Each time interval is considered as a cluster by its own. For each time interval, we compute three properties that represent the cluster centroid of the test time interval: (1) the sensor feature vector; (2) the time reference; (3) the sensor structure. The sensor feature vector is computed as described in Section 5. The time reference is computed using Algorithm 4. Finally, the sensor structure is computed using Algorithm 14, but the input will be the sensor feature vector ${\vec{f}}_{i}^{s}$.

In order to classify the time interval in the testing day to one of the corresponding clusters of the training days, we compute the times’ reference similarity as in Equation (14), the sensors’ time duration similarity as in Equation (15), and the sensors’ structure similarity as in Equation (16). Finally, the group-average link is computed as in Equation (17) between the test time interval and one of the clusters in the training set. This process is repeated between the test time interval, and all the clusters in the training set. The test time interval is classified to the cluster with the best similarity value. The cluster label will be the label to be assigned to the test time interval. We will refer to this approach as classification by cluster. Table 4 summarizes the tuned parameters of our framework. The parameters values were found to be acceptable based on several runs of our experiments.

In the second approach, we use the cluster data points and the semantic label associated with each cluster to build a model, in order to classify the label of the test time interval. Each data point has three properties: the sensor feature vector, the time reference, and the sensor structure. These properties are used as inputs to train a model, and the semantic label of each data point is used as the output of the model. We chose the perceptron classifier to build the model. Other classifiers were tested, but the perceptron classifier gave the best performance among them. We will refer to this approach as classification by model.

### 8.3. Evaluation Criteria

We are mainly interested in evaluating the performances of our methods on the routines including and excluding the “other activity” class. Therefore, we calculated the classification accuracy and the F-score as measures for the validation. The predefined activity classification accuracy shows the percentage of correctly classified instances:

(19) $$Accuracy=\sum_{i=1}^{|\xi^{s}|}\frac{TP_{\xi_{i}^{s}}}{N_{\xi_{i}^{s}}},$$

where $\xi_{i}^{s}$ is the classified activity, $N_{\xi_{i}^{s}}$ is the total number of the time intervals associated with a classified activity $\xi_{i}^{s}$ (such as “sleep”), $TP_{\xi_{i}^{s}}$ is the number of correctly classified windows for this predefined activity, and $|\xi^{s}|$ is the total number of predefined activities. The F-score for activity $\xi_{i}^{s}$ is computed as follows:

(20) $$F-score=2\times \frac{P\times R}{P+R},$$

where *P* and *R* represent the precision and the recall for activity $\xi_{i}^{s}$. The F-score is favored over accuracy when we have an imbalanced data set due to its inherent capacity to measure a recognition method performance on a multi-class classification problem, and because it is adapted for the class distributions of the ground truth and the classified activity labels. Additionally, it is commonly used as an evaluation measure in smart home settings for evaluating the performances of human activity recognition methods as in [46,63,64]. Therefore, we follow a similar approach by using the F-score measure.

### 8.4. Results and Discussion

We conducted four sets of experiments to evaluate the effectiveness of the methods presented in this paper. In the first experiment, we evaluated the pattern discovery of our clustering methods in Section 6. In the second experiment, we evaluated our framework using the sensor data while excluding the “other activity” class. In the third experiment, the “other activity” class was incorporated during the evaluation of our framework. In the last experiment, we evaluated and compared our framework against other state-of-the-art methods.

#### 8.4.1. Pattern Discovery Evaluation

**Figure 21:**
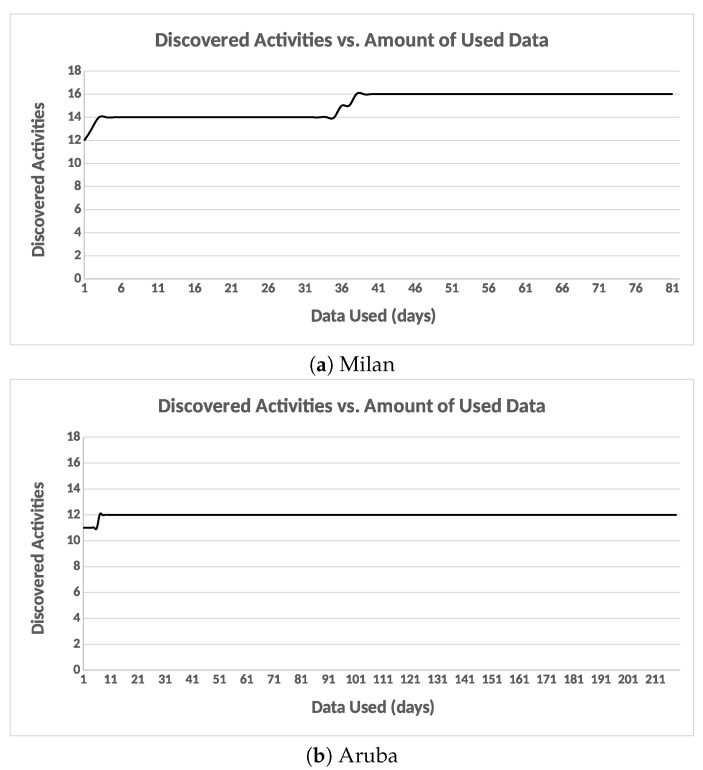
Number of the unique activities detected vs. the size of data.

First, we analyze the high-level processing step, which is composed of “intra-day pattern discovery”, “inter-day pattern discovery”, and “cluster refinement”. The high-level processing step was varied, and capable of detecting most of the predefined routines of interest. On the “Milan” data set, it discovered 16 out of 16 activities. On the “Aruba” data set, it was able to discover all of the 12 activities. Some of the patterns that were not detected instantly are in fact very challenging to find and in several situations less recurrent. For example, the “Eve_Meds” is not linked to any particular unique sensor. Not only that, several similar patterns are combined with each other, as they employ the exact same group of sensors, such as “Morning_Meds” and “Kitchen_Activity” on the “Milan” data set. Moreover, some of the routines are detected more than once in the format of varied patterns, as the routine can be carried out in different ways, activating various sensors. Figure 21a,b shows the number of unique detected activities by the high-level processing step. One can clearly see that the high-level processing step is capable of detecting a large number of unique activities. These figures present the size of data for reaching a particular level of accuracy as well.

**Figure 22:**
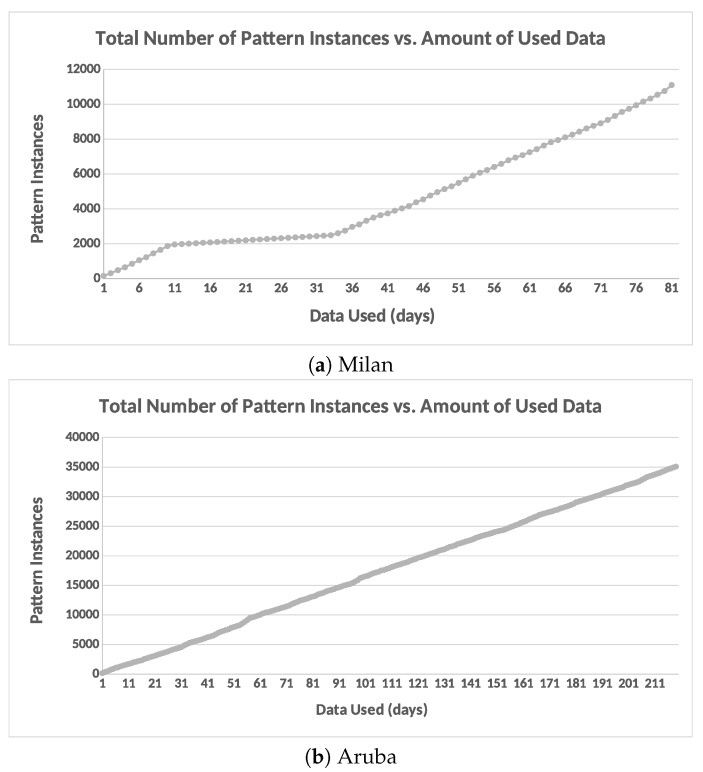
Number of pattern occurrences vs. the size of data.

**Figure 23:**
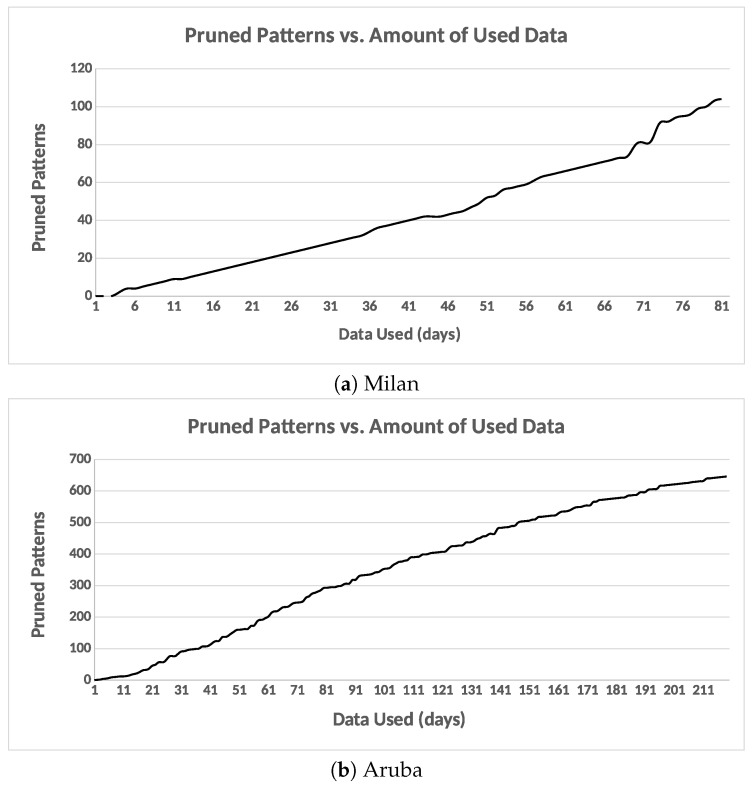
Number of trimmed patterns vs. the size of data.

Figure 22a,b shows the number of detected patterns occurrences for the high-level processing step, while Figure 23a,b shows the number of trimmed occurrences. In some cases the high-level processing step produces more pattern occurrences and in general more patterns; it also trims pattern occurrences, while still detecting more unique patterns (activities).

The clusters were further refined using our cluster refinement approach described in Section 6. By using the externally provided labels, the purity of the clusters were measured with respect to the coherence and cohesion of their differences using Equation (21). The cluster purity is defined as the percentage of the total number of data points that were classified correctly, in the unit range [0…1]. The purity is computed as follows:

(21) $$Purity=\frac{1}{N^{*}}\sum_{i=1}^{|{\hat{\vec{c}}}_{1}^{s}|}max_{j}\left|{\hat{o}}_{i}^{s}\cap \xi_{j}^{s}\right|,$$

where $N^{*}$ is the number of data points, $|{\hat{\vec{c}}}_{1}^{s}|$ is the number of clusters, ${\hat{o}}_{i}^{s}$ is a cluster in ${\hat{\vec{c}}}_{1}^{s}$, and $\xi_{j}^{s}$ is the classification that has the max count for cluster ${\hat{o}}_{i}^{s}$. We use the ground truth classification of those objects as the measure of assignment correctness, however to do so we must know, which cluster ${\hat{o}}_{i}^{s}$ maps to which ground truth classification $\xi_{i}^{s}$. If it was 100% accurate, then each ${\hat{o}}_{o}^{s}$ would map to exactly 1, but in reality our cluster ${\hat{o}}_{i}^{s}$ contains some points whose ground truths classified them as several other classifications. Naturally then we can see that the highest clustering quality will be obtained by using the ${\hat{o}}_{i}^{s}$ to $\xi_{i}^{s}$ mapping, which has the highest number of correct classifications; i.e., ${\hat{o}}_{i}^{s}\cap \xi_{i}^{s}$. That is where the max comes from in the equation. To calculate purity, a confusion matrix is created. This can be done by looping through each cluster ${\hat{o}}_{i}^{s}$ and counting how many objects were classified as each class $\xi_{i}^{s}$.

The purity of the “Milan” data set was 0.882 in our experiments, while purity for the “Aruba” data set was 0.948. By paying a closer look at the data and analyzing it, we found that the patterns in the “Milan” data set were considerably infrequent. For that reason, the most similar patterns were merged together into one cluster; e.g., taking medication and kitchen activity, which normally occur at around the same hour and the same place (in this occasion, the kitchen). Our clustering methods are able to achieve an average purity level of 0.915. On the other hand, the COM [5] method achieved an average purity level of 0.830 on two private data sets. This reveals that our clustering methods can better identify and group similar patterns to the same clusters than the COM [5] method.

#### 8.4.2. Excluding “Other Activity” Class Evaluation

After forming the clusters, and assigning a semantic label for each cluster, we used two approaches; namely, classification by cluster and classification by model to classify the time intervals of a test day to the predefined activities. In the second experiment, the framework was trained on data without considering the “other activity” class. This class is included in the third experiment to evaluate the framework. Table 5 shows the accuracy and F-score of the two classification approaches without considering the “other activity” class using the “Aruba” and “Milan” data sets. The classification by model approach achieved an average accuracy and F-score of 95.53% and 95.78%. The classification by cluster approach achieved an average accuracy and F-score of 96.59% and 96.48%. On average, the classification by cluster has an increased performance of 1% over the classification by model.

**Figure 24:**
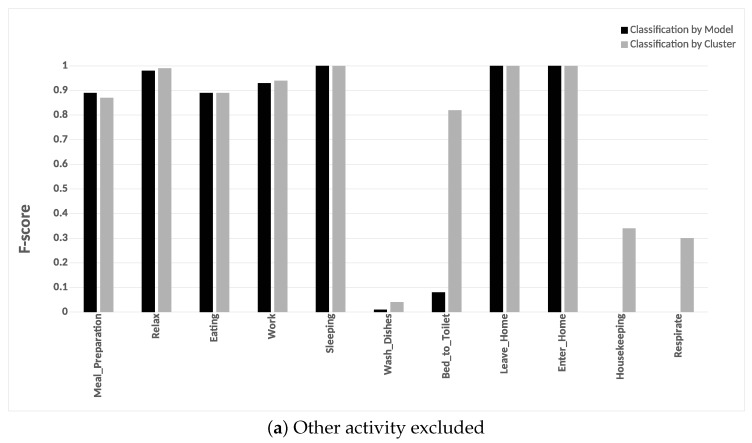

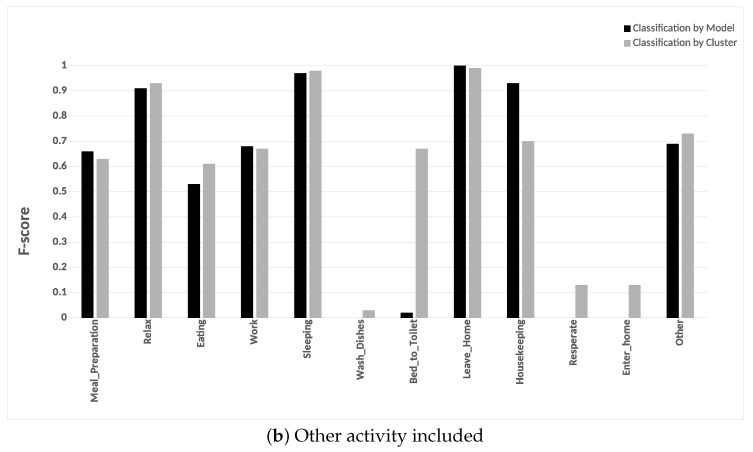
F-scores for each activity for Aruba data set as achieved by the different classification methods.

**Figure 25:**
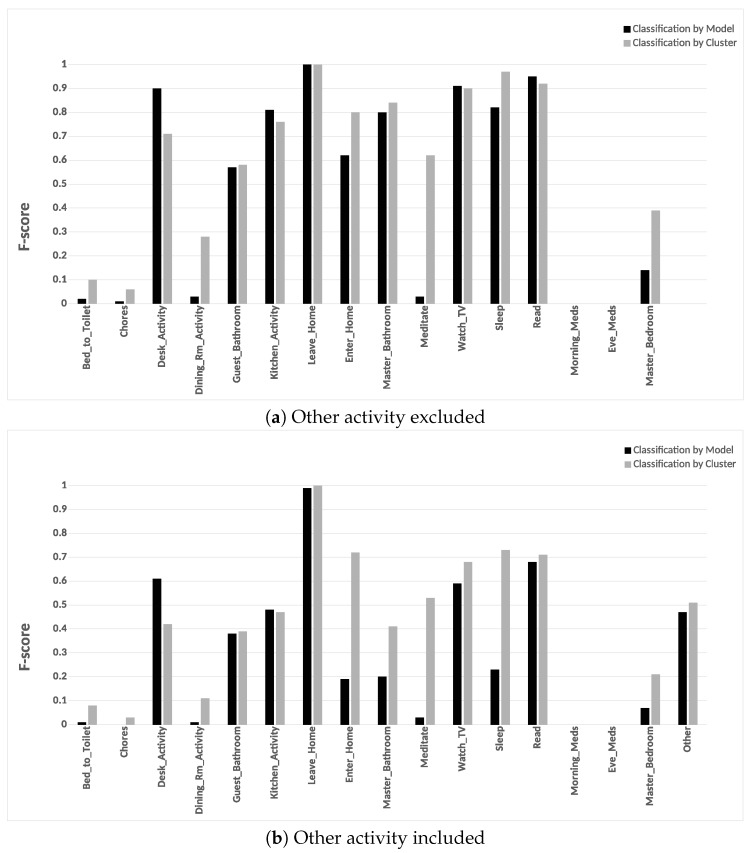
F-scores for each activity for the Milan data set as achieved by the different classification methods.

Furthermore, Figure 24a and Figure 25a show the F-scores of the individual activities for “Aruba” and “Milan” data sets as obtained by the different classification approaches. It can be noted from these figures that due to larger recurrence in “Aruba” data set, it is easier to track and recognize activities. From the “Aruba” and “Milan” data sets, there are some activities like “sleep”, “relax”, “read”, “Watch_TV”, and “Leave_Home” that are classified comparatively better than the other activities. These activities are executed in clear places and/or fixed time through the day. For example, the activity “work” takes place in the evening in the office location of the “Aruba” data set. Each data set has a certain sensor that tracks the motion in the office location, and thus, behaves as a strong indicator for the activity.

In Figure 24a, some of the activities such as “eating”, “Wash_Dishes”, “Bed_to_Toilet”, and “housekeeping” are detected with low accuracy, because there is some confusion between similar activities such as “Meal_Preparation” and “Wash_Dishes”, which occur at exact hours and exact places. Additionally, the “housekeeping” activity occurs in very rare occasions in comparison to other activities; and even if it has enough frequency in the data, because of its unorganized nature and the fact that it takes place all over the apartment, it is very hard to detect. Therefore, it is mistaken with other activities, such as “relax” or “work”. The “housekeeping” activity happens every 2–4 weeks in the “Aruba” data set, and it is not associated with any specific sensor. The “housekeeping” activity required the person to move around different locations, and activate many sensors. Therefore, it is hard to capture this activity using PIR sensors only. Sensorizing “housekeeping” tools such as broom, duster, and dustpan will definitely yield better results in terms of detecting this activity accurately. Because the “housekeeping” activity will have its own sensor data pattern and signature. This will enable our methods to find distinct clusters for it. The “housekeeping” activity was not discovered in the first place in DVSM [51] and COM [5] methods, while our method was able to discover this activity given the previously mentioned challenges. The rest of activities are detected with a high accuracy. It can be noted that the classification by cluster outperforms the classification by model in detecting these activities.

**Figure 26:**
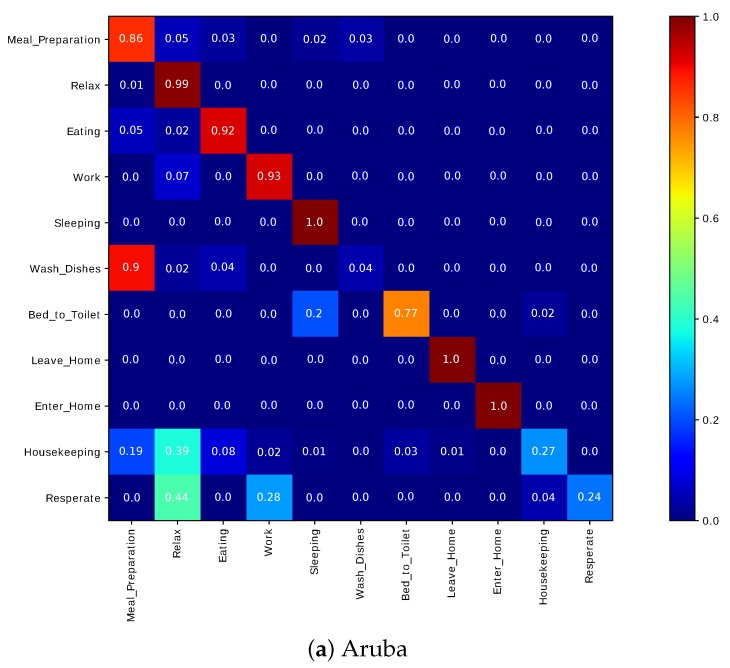

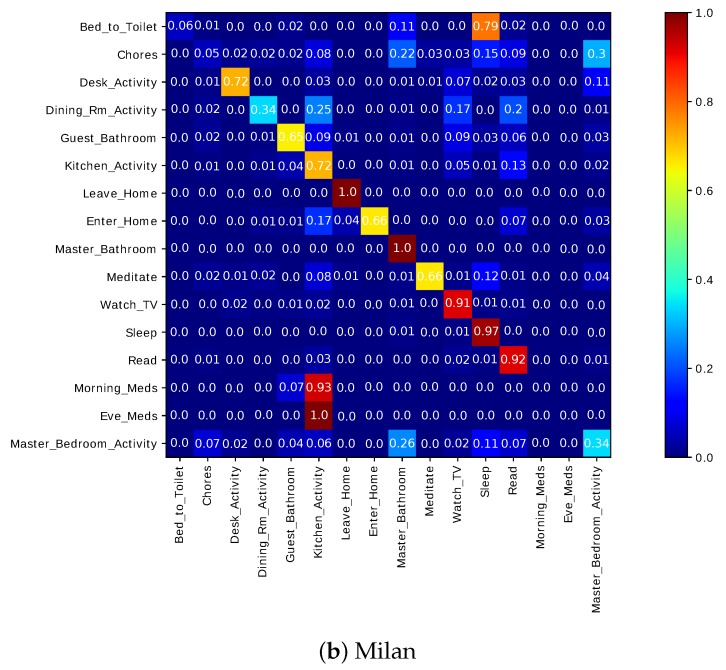
Confusion Matrix for Aruba and Milan data sets as obtained by the classification by cluster approach without considering “other activity” class.

On “Milan” data set, both approaches achieve a lower accuracy as shown in Figure 25a. This can be attributed to the fact that some activities such as “Kitchen_Activity”, “Morning_Meds”, and “Eve_Meds” occur at similar location as in the kitchen. Detecting “Morning_Meds” and “Eve_Meds” activities can be further improved by equipping the kitchen cabinet containing a medicine dispenser with vibration sensors, accelerometers, and RFID sensors. These activities will have their own sensor activation patterns. Our methods can then group them accurately into separate clusters, and not with the same clusters of “Kitchen_Activity”. Other activities such as “Bed_to_Toilet” might be mistaken with another activity such as “sleep” because these activities are less present in the data set. Figure 26 shows the confusion matrix of the classification by cluster approach for the two data sets without considering the “other activity” class.

#### 8.4.3. Containing “Other Activity” Class Evaluation

In this experiment, the “other activity” class is included. The results are summarized in Table 6. By comparing the results obtained in the first experiment (Table 5) with the one obtained by this experiment (Table 6), the average classification accuracy and F-score of the classification by model approach drop about 21% and 22%. Similarly, the average accuracy and F-score of the classification by cluster approach drop about 16% and 17%. To further analysis this significant drop, we plotted the individual F-score for each activity; that is summarized in Figure 24b and Figure 25b. From Figure 24b, “Wash_Dishes” has the most significant drop with an F-score of 7%, while several activities such as “resperate” and “Enter_Home”, have the second most significant drop with an F-score of 13%. The remaining activities did not have a significant decrease in their F-scores. Similar observations can be concluded from Figure 25b; several activities such as “sleep”, “read”, “Watch_TV”, “meditate”, “Enter_Home”, and “Leave_Home” did not have significant drops in their F-scores, while we can see a noticeable drop in the F-scores of the remaining activities.

**Figure 27:**
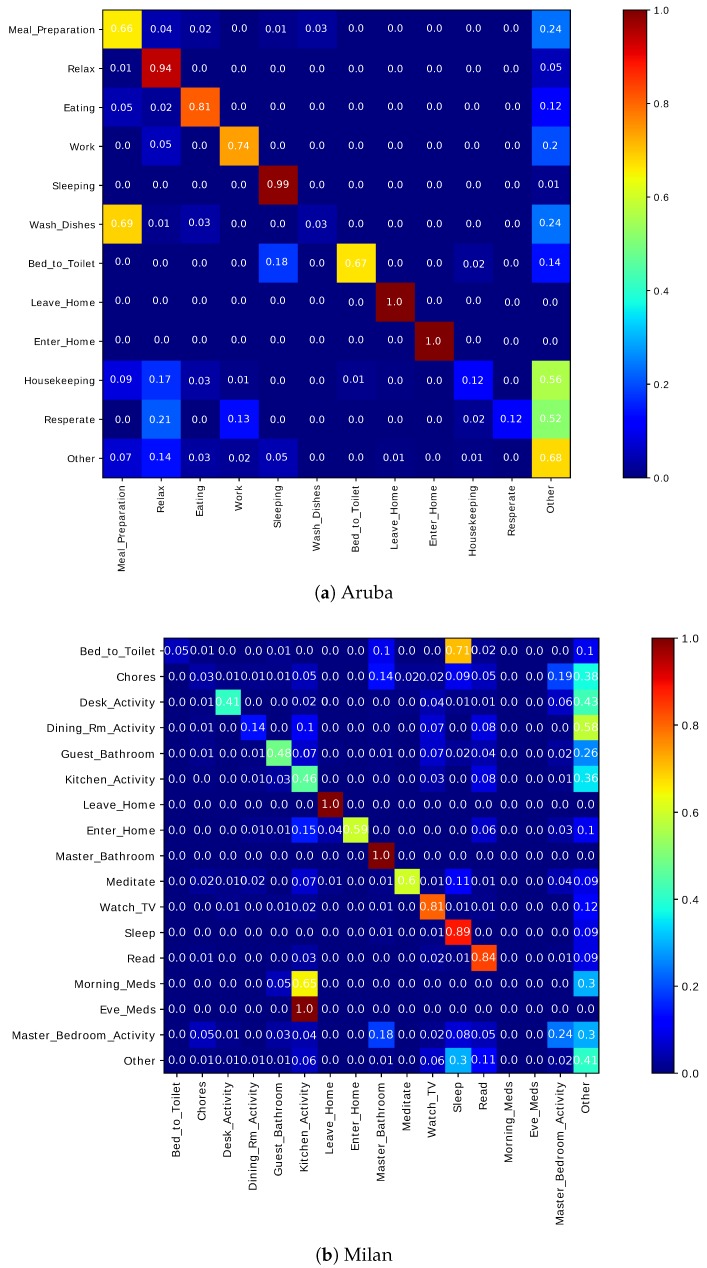
Confusion Matrix for Aruba and Milan data sets as obtained by the classification by cluster approach considering “other activity” class.

As the graphs and tables illustrate, the accuracy performance degrades when the non-labeled data is included in the analysis. There are two reasons for this change in performance. First, the “other activity” class dominates the data; thus many data points that belong to predefined activities are misclassified as “other activity” class (this can be seen in the confusion matrix graphs in Figure 26 and Figure 27). Second, the “other activity” class itself represents a number of different activities, transitions, and movement patterns. As a result, it is difficult to characterize this complex class and difficult to separate it from the other activity classes.

#### 8.4.4. Comparison to Other Methods

Finally, we compare our approach against other four approaches on “Aruba” and “Milan” data sets using F-score as shown in Table 7. The “GA” [34], “semi-supervised” [32], and kernel fusion [22] approaches did not include the “other activity” class in their experiments and analysis, but the “MkRENN” approach did [25]; the authors performed their experiments two times by excluding and including the “other activity” class. From Table 7, our approach achieves the best performance on “Aruba” data set in comparison to the other approaches when the “other activity” class is excluded. MkRENN [25] achieved an F-score of 53.78% when the “other activity” class was included, and our approach achieved an F-score of 89.33%. Our approach still outperforms the MkRENN approach even when the “other activity” class is included. On the “Milan” data set, our approach scored an F-score of 94.32%, which is slightly better than the result of the “Kernel Fusion” approach [22] with an F-score of 94.11%.

**Figure 28:**
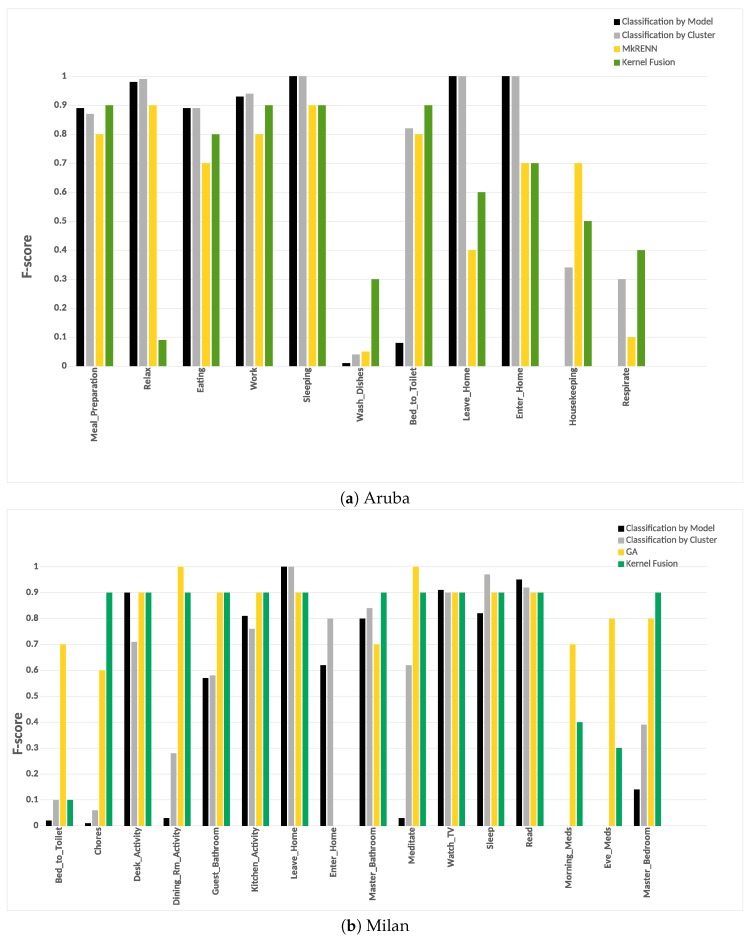
Comparing the F-scores for the individual activities on Aruba and Milan data sets as obtained by the different approaches.

We provide a more detailed comparison in Figure 28 between our two classification approaches and the listed approaches in Table 7 on “Aruba” and “Milan” data sets. We show the F-score measure of each activity obtained by each method. “Semi-supervised [32]” method is excluded from this comparison since the authors did not report the F-score measures of each activity in their work. In Figure 28a, our two proposed recognition methods: “classification by model”, and “classification by cluster” perform better than “MkRENN” and “kernel fusion” methods on detecting: “relax”, “eating”, “work”, “sleeping”, “Leave_Home”, and “Enter_Home” activities. While, our methods deliver close F-score results to the other methods on detecting: “Meal_Preparation”, and “Bed_to_Toilet” activities. The good performance of our methods is attributed to: (1) the use of multiple time interval lengths to extract the time duration features around the detection areas of sensors and locations; (2) our clustering algorithms use several data point properties such as the sensor structure, the time of the sensor feature, and the time duration to measure the similarity between clusters; (3) our novel approach of extracting “Leave_Home” and “Enter_Home” features helped our clustering algorithms to group these activities accurately with distinct data point properties; (4) the introduction of several clustering steps in the high-level processing stage, where different similarity measures such as exponential decay model and EMD are used in order to refine the patterns into a more compressed and defined cluster characterizations; (5) the use of the location as a cue in our feature extraction; and (6) from Table 3, the aforementioned activities occurred enough times in the “Aruba” data set for a long period of time, and this gave our methods sufficient data to explore and discover the different pattern variations of these activities. The “Kernel Fusion” and “MkRENN” methods did not perform well, because these methods could not capture the underlying variations in these long-term patterns, since the sequence patterns are disrupted by irrelevant events, and have many variations in terms of step ordering or duration.

All the methods including ours were not able to detect certain activities with a good accuracy: “Wash_Dishes”, and “respirate” activities. “MkRENN” and “GA” perform better than our methods in detecting “housekeeping” activity. This can be attributed to the following: (1) from Table 3, these activities did not occur enough times in the data set, which made them difficult to be discovered or recognized; (2) our methods may assign some of these activities to a wrong but similar cluster, this can be seen in activities such as “Wash_Dishes” and “Meal_Preparation”, because the dish cleaning and meal preparation tasks happen in the same location, and trigger the same set of sensors, so in many cases they were clustered together; and (3) our clustering algorithms considered the “housekeeping” pattern to be variations of “Relax” and “Meal_Preparation” patterns.

In Figure 28b, our two proposed recognition methods: “classification by model”, and “classification by cluster” perform better than “GA” and “kernel fusion” methods on detecting: “Leave_Home”, “Enter_Home”, “sleep”, and “read”. Additionally, our methods deliver close F-score results for the other methods on detecting: “Desk’_Activity”, “Kitchen_Activity”, “Bed_to_Toilet”, “Master_Bathroom”, and “Watch_TV”. The reasons of the good performance of our methods is similar to the “Aruba” data set. The other methods could not detect some of the activities accurately such as “Leave_Home”, and “Enter_Home” due to the poor feature representation, in which they were used to train these models.

Our methods detected some activities less accurately than the other methods: “meditate”, and “Guest_Bathroom”. Our methods had challenges in discovering and recognizing several activities accurately: “chores”, “Dining_Rm_Activity”, “Morning_Meds”, “Eve_Meds”, and “Master_Bedroom”. This can be attributed to the following: (1) From Table 3, these activities have less frequency than the other activities; therefore our clustering algorithm considered them as variations of the other patterns. For example, “Morning_Meds”, and “Eve_Meds” have 19, and 41 occurrences respectively, while “Kitchen_Activity” has 554 occurrences, since “Morning_Meds”, “Eve_Meds”, and “Kitchen_Activity” occur in the same place, and trigger the same set of sensors, our clustering algorithms assign “Morning_Meds” and “Eve_Meds” patterns to the dominant cluster “Kitchen_Activity”. To an extent this highlights the fact that “Morning_Meds” and “Eve_Meds” are in fact very similar and could possibly be monitored together as a group. The same observation can be seen in case of “chores”, and “Dining_Rm_Activity” activities. (2) Our methods were able to detect “Mediate” activity to a certain extent, given the low number of occurrences (17), but some instances of this activity have been confused with other patterns such as “sleep” due to the similar sensor structure between “Mediate” and the other activities. This confusion can be noted as well in “Master_Bedroom” where it resembles similar sensor and location information to “sleep” and “Master_Bathroom” activities. (3) One major advantage in “Kernel Fusion” and “GA” methods is that they rely on training different kernel functions and classifiers; then they fuse the individual results on a decision level. This technique greatly boosts the accuracy of detecting these activities, given their low number of occurrences.

The one can observe that our methods can perform well and better than the other approaches, when the activities have enough occurrences and repetitions, and this can be seen in the case of the “Aruba” data set. Our methods in this case can deal with different variation of the same pattern. While the other methods fail to learn the relationship between the attributes of the sensors features for the different variation of the same pattern, and the target class. On the other hand, our methods struggle with discovering imbalanced activities or the activities with insufficient occurrences and repetitions. This can be seen in the case of the “Milan” data set. Our methods in this case may group some instances of these activities to the nearest dominant cluster, where they share similar sensor structure, time references, and sensors duration. The other methods rely on an ensemble of classifiers to learn the mapping from the sensor data to the target class. This approach works well with imbalanced data.

Sensorizing tools, and adding more environmental sensors will definitely help our clustering algorithms to identify these activities accurately.

## 9. Conclusions

In this paper, we proposed a framework for activity recognition from the motion sensor data in smart homes. We used a time-based windowing approach to compute features at different granularity levels. Then, we introduced an unsupervised method for discovering activities from a network of motion detectors in a smart home setting without the need for labeled training data. First, we presented an intra-day clustering algorithm to find frequent sequential patterns within a day. As a second step, we presented an inter-day clustering algorithm to find the common frequent patterns between days. Furthermore, we refined the patterns to have a more compressed and defined cluster characterizations. Finally, the clusters were given semantic labels in order to track the occurrences of the future activities.

We discussed two approaches for recognizing activities using the final set of the clusters, where they are used to recognize the occurrences of the future activities from a window of sensor events. The first approach used a model to classify the labels, while the second approach used to measure the similarity between the clusters centroids, and the test time interval to determine the cluster with the best similarity value, where the semantic label of that cluster is assigned.

Our framework was evaluated on two public data sets captured in real-life settings from two apartments during a seven-month and a three-month periods. Experiments on the two real-world data sets revealed that the classification by cluster achieved a higher F-score and accuracy. Our experiments included evaluating the two classification approaches with and without “other activity” class. Finally, our approach achieved a higher F-score than the other approaches.

When the “other activity” class is included, there was a drop in the F-score of the individual activities of our approach, this drop was due to identifying the “other activity” class as known activities. This shows the ability of our framework to discover patterns in the “other activity” class similar to the predefined activities. One of our future goal work is to evaluate the robustness of the proposed framework on data being captured from various smart homes with more than one resident.

## Figures and Tables

**Table 1:** List of location labels used for processing.

Identifier	Location Description
0	Hallway
1	Kitchen
2	Dining area
3	Living room
4	Main bedroom
5	Guest bedroom
6	Bathroom
7	Office
8	Outside of apartment

**Table 2:** List of the transitions and the conditions to update $d_{p}^{s_{N+1}}$ and $d_{p}^{s_{N+2}}$, where these sensor feature values will represent the ENTER_HOME and LEAVE_HOME activities.

Transition	Condition	Update
T1	$b_{p}^{s}\geq h_{p}^{s}\wedge d_{p-1}^{s_{N+1}}=0\wedge d_{p-1}^{s_{N+2}}=0$	$d_{p}^{s_{N+1}}=0$
		$d_{p}^{s_{N+2}}=0$
T2	$b_{p}^{s}\geq h_{p}^{s}\wedge d_{p-1}^{s_{N+1}}=0\wedge d_{p-1}^{s_{N+2}}=2\psi_{s}$	$d_{p}^{s_{N+1}}=0$
		$d_{p}^{s_{N+2}}=0$
T3	$h_{p}^{s}\geq b_{p}^{s}\wedge d_{p-1}^{s_{N+1}}=0\wedge d_{p-1}^{s_{N+2}}=0$	$d_{p}^{s_{N+1}}=0$
		$d_{p}^{s_{N+2}}=2\psi_{s}$
T4	$h_{p}^{s}\geq b_{p}^{s}\wedge d_{p-1}^{s_{N+1}}=0\wedge d_{p-1}^{s_{N+2}}=2\psi_{s}$	$d_{p}^{s_{N+1}}=0$
		$d_{p}^{s_{N+2}}=2\psi_{s}$
T5	$b_{p}^{s}\geq h_{p}^{s}\wedge d_{p-1}^{s_{N+1}}=0\wedge d_{p-1}^{s_{N+2}}=2\psi_{s}$	$d_{p}^{s_{N+1}}=2\psi_{s}$
		$d_{p}^{s_{N+2}}=0$
T6	$h_{p}^{s}\geq b_{p}^{s}\wedge d_{p-1}^{s_{N+1}}=2\psi_{s}\wedge d_{p-1}^{s_{N+2}}=0$	$d_{p}^{s_{N+1}}=0$
		$d_{p}^{s_{N+2}}=2\psi_{s}$
T7	$b_{p}^{s}\geq h_{p}^{s}\wedge d_{p-1}^{s_{N+1}}=2\psi_{s}\wedge d_{p-1}^{s_{N+2}}=0$	$d_{p}^{s_{N+1}}=0$
		$d_{p}^{s_{N+2}}=0$

**Table 3:** Characteristics of the annotated activities of CASAS smart home data sets.

Apartment 1				Apartment 2			
Id	Activity	# of Sensor Events	Activity Count	Id	Activity	# of Sensor Events	Activity Count
1	Meal Preparation	299,300	1606	1	Bed to Toilet	1255	89
2	Bed to Toilet	1483	157	2	Sleeping	22,172	96
3	Relax	387,851	2919	3	Leave Home	4946	214
4	Sleeping	63,792	401	4	Watch TV	23,688	114
5	Eating	19,568	257	5	Chores	7587	23
6	Enter Home	2041	431	6	Desk Activity	7628	54
7	Housekeeping	11,010	33	7	Dining Rm Act	4295	22
8	Leave Home	1954	431	8	Evening Medicines	250	19
9	Respirate	571	6	9	Guest Bathroom	10,601	330
10	Wash Dishes	10,682	65	10	Kitchen Activity	128,942	554
11	Work	17,637	171	11	Master Bathroom	15,071	306
12	Other Activity	903,669	-	12	Master Bedroom	27,337	117
				13	Meditate	1315	17
				14	Morning Medicines	1023	41
				15	Read	50,281	314
				16	Other Activity	21,774	-

**Table 4:** Tuned parameters of the proposed framework.

Parameters	Values
Sensor time interval length $\psi_{s}$	60 s
Location time interval length $\psi_{l}$	120 s
Degree of location similarity $\lambda_{l}$	$2^{-6}$
Degree of sensor similarity $\lambda_{s}$	$2^{-5}$
EMD similarity threshold β	0.99
Cluster refinement threshold ζ	0.99

**Table 5:** Results of the two classification approaches without considering “other activity” class.

Approaches	Aruba		Milan	
	Accuracy	F-Score	Accuracy	F-Score
Classification by Cluster	98.66%	98.64%	94.52%	94.32%
Classification by Model	98.00%	98.28%	93.05%	93.27%

**Table 6:** Results of the two classification approaches considering “other activity” class.

Approaches	Aruba		Milan	
	Accuracy	F-Score	Accuracy	F-Score
Classification by Cluster	89.48%	89.33%	71.88%	70.68%
Classification by Model	87.64%	87.88%	60.76%	59.19%

**Table 7:** F-score comparisons to other approaches on Aruba and Milan data sets.

Approaches	Aruba	Milan
Ours	98.64%	94.32%
MkRENN [25]	66.26%	-
GA [34]	-	85.73%
Semi-Supervised [32]	-	78.24%
Kernel Fusion [22]	92.70%	94.11%
